# Dehydrogenative Double C−H Bond Activation in a Germylene‐Rhodium Complex**

**DOI:** 10.1002/chem.202102529

**Published:** 2021-10-27

**Authors:** Sonia Bajo, María M. Alcaide, Joaquín López‐Serrano, Jesús Campos

**Affiliations:** ^1^ Instituto de Investigaciones Químicas (IIQ) Departamento de Química Inorgánica and Centro de Innovación en Química Avanzada (ORFEO-CINQA) Consejo Superior de Investigaciones Científicas (CSIC) and University of Sevilla 1Avenida Américo Vespucio 49 41092 Sevilla Spain

**Keywords:** C−H bond activation, chemical cooperation, germylene, metallotetrylene, tetrylene

## Abstract

Transition metal tetrylene complexes offer great opportunities for molecular cooperation due to the ambiphilic character of the group 14 element. Here we focus on the coordination of germylene [(Ar^Mes2^)_2_Ge :] (Ar^Mes^=C_6_H_3_‐2,6‐(C_6_H_2_‐2,4,6‐Me_3_)_2_) to [RhCl(COD)]_2_ (COD=1,5‐cyclooctadiene), which yields a neutral germyl complex in which the rhodium center exhibits both *η* ^6^‐ and *η* ^2^‐coordination to two mesityl rings in an unusual pincer‐type structure. Chloride abstraction from this species triggers a singular dehydrogenative double C−H bond activation across the Ge/Rh motif. We have isolated and fully characterized three rhodium‐germyl species associated to three C−H cleavage events along this process. The reaction mechanism has been further investigated by computational means, supporting the key cooperative action of rhodium and germanium centers.

## Graphical abstract

**Transition metal tetrylenes** offer new ways for chemical cooperation with great prospects for catalysis. Here we report on an unusual naked rhodium(I) site that is embraced by a bulky germylene in a pincer‐like fashion. Cooperation across the Rh−Ge motif is illustrated by up to three C−H activation events, along with hydride and formal alkyl migrations between the two sites.

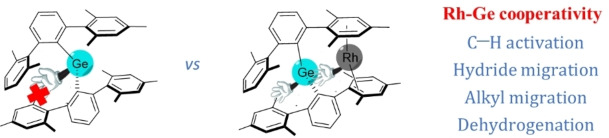

## Introduction

Transition metal complexes bearing ambiphilic ligands that combine both electron donor and acceptor groups have attracted a great deal of attention in recent times.^[1]^ In this regard, heavier tetrylenes (: ER_2_; E=Si, Ge, Sn, Pb) offer unique opportunities as single‐site ambiphiles (σ‐donating lone pair and empty p orbital), revealing unusual coordination modes and reactivity.^[2]^ Moreover, they represent the prospects of new avenues for transition metal/P‐element cooperation.[^3^, ^4^] However, the coordination chemistry of tetrylenes remains considerably less explored than their lighter carbene congeners, in no little part due to reduced stability. To overcome this limitation base‐stabilized tetrylenes have been explored and their complexes have found relevance in catalysis.^[5]^ Nonetheless, quenching their Z‐type character by inter‐ or intramolecular bases hampers their potential to actively cooperate with the transition metal in bond activation processes.

Steric shielding around the tetrel site has also been widely exploited as a strategy to provide kinetic stabilization, terphenyl (C_6_H_3_‐2,6‐Ar_2_) substituents being among the preferred choice.^[6]^ In fact, coordination of terphenyl‐stabilized tetrylenes to transition metals has already provided compelling results,^[7]^ revealing the tunable donor/acceptor nature of the group 14 element^[8]^ and the realization of its highly dynamic binding capacity.^[9]^ For instance, the interconversion with tetrylidyne (M≡E−R) and tetryl (M−ER_3_) forms drastically modify the bonding with the transition metal and its stereoelectronic properties, producing reactive unsaturated sites amenable for divergent reactivity.^[10]^ In this study, we report the formation of a rhodium germylene/germyl complex based on the bis‐terphenyl [(Ar^Mes2^)_2_Ge :] (Ar^Mes^=C_6_H_3_‐2,6‐(C_6_H_2_‐2,4,6‐Me_3_)_2_)^[11]^ that promotes a unique dehydrogenative double C−H bond activation process in which the germanium center reversibly rearranges from germylene to germyl forming Ge−Cl, Ge−H and Ge−C bonds in concert with the rhodium site.

## Results and Discussion

**Scheme 1:**
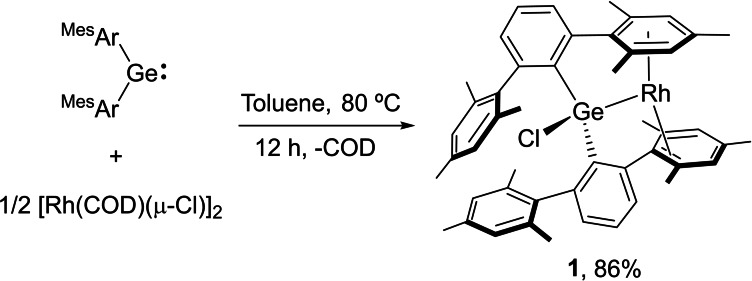
Synthesis of germyl rhodium complex **1**.

**Figure 1:**
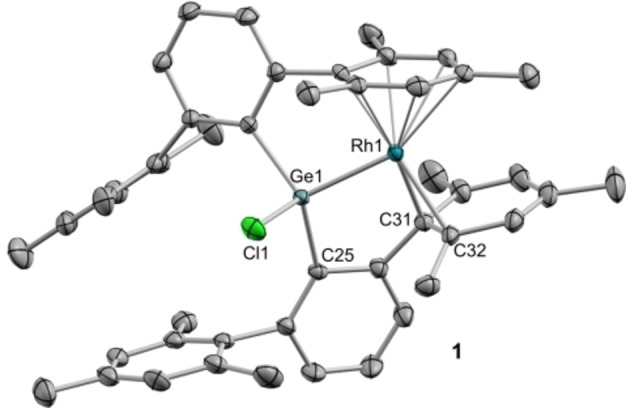
ORTEP diagram of **1**. Hydrogen atoms are excluded for clarity, and thermal ellipsoids are set at 50 % probability.

Heating an equimolar toluene solution of [RhCl(COD)]_2_ (COD=1,5‐cyclooctadiene) and [(Ar^Mes2^)_2_Ge :] at 80 °C for twelve hours afforded the formation of germyl rhodium complex **1**, which precipitated from the reaction media as a dark orange solid in 86 % yield (Scheme 1). The release of COD, clearly identified by ^1^H NMR, is accompanied by *η* ^6^‐coordination of the π‐system of one of the flanking aryl rings of a terphenyl substituent.[^9a^, ^9d^, ^12^] This is consistent with a somewhat deshielded ^1^H NMR resonance at 5.16 ppm (2 H) that contrasts with the corresponding resonance in [(Ar^Mes2^)_2_Ge :] found at 6.76 ppm. The associated ^13^C{^1^H} NMR signal resonates at 119.9 ppm (c.f. 129.0 ppm in [(Ar^Mes2^)_2_Ge :]) and exhibits scalar coupling to ^103^Rh (d, ^1^ *J*_CRh_=9 Hz), in line with the proposed coordination. The molecular formulation of **1** was ascertained by X‐ray diffraction studies (Figure 1), evidencing insertion of the germylene into the Rh−Cl bond, as previously observed in other rhodium/germylene systems.^[13]^ Besides, the rhodium center is *η* ^2^‐coordinated to a lateral ring of the alternate terphenyl fragment (Rh1−C31=2.153(2) and Rh1−C32=2.194(2) Å),^[14]^ thus resembling an unusual type of pincer‐type coordination of the germyl moiety. Despite seemingly a simple process, these two aryl rings do not exchange at the NMR time scale even upon heating the probe to 80 °C.

**Scheme 2:**
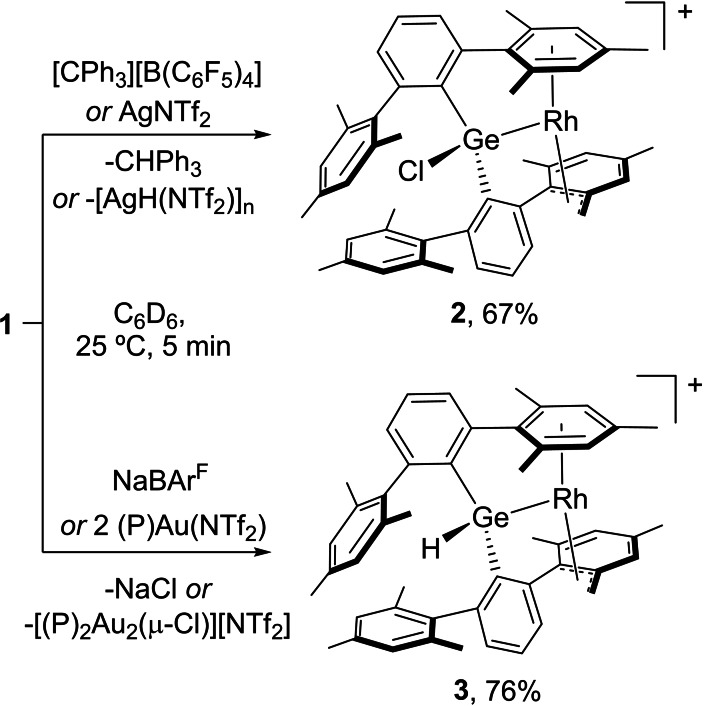
Synthesis of compounds **2** and **3** from germyl‐rhodium **1** by hydride and chloride abstraction, respectively.

To investigate bond activation processes through Ge/Rh bimetallic cooperation^[15]^ we first targeted chloride abstraction from **1**. Addition of AgNTf_2_ (NTf_2_=triflimidate=[N(SO_2_CF_3_)_2_]^−^) surprisingly led to the withdrawal of a hydride, inferred by a distinctive ^1^H NMR resonance at 0.87 ppm (t, ^1^ *J*_HAg_=7 Hz) attributable to [AgH(NTf_2_)]_n_,^[16]^ readily generating germyl‐rhodium **2** in good yields (Scheme 2). In fact, an analogous process instantly takes place by treatment of **1** with trityl salt [CPh_3_][B(C_6_F_5_)_4_], with concomitant formation of CHPh_3_. The activation of one benzylic C−H bond originates a characteristic AB system in the ^1^H NMR spectra due to the diastereotopic Rh‐CH_2_ protons, with slightly broad signals at 3.62 and 1.31 ppm. Their corresponding ^13^C{^1^H} NMR peak resonates at 42.7 ppm (d, ^1^ *J*_CRh_=12 Hz).

To our delight, treatment of **1** with NaBAr^F^ immediately effects the initially targeted chloride abstraction. The resulting compound **3** is spectroscopically similar to chloride‐containing **2**. Thus, an analogous AB pattern is present, but accompanied by an additional resonance at 6.02 due to a Ge−H terminus. Interestingly, addition of two equivalents of (PMe_2_Ar^Dipp2^)Au(NTf_2_)^[17]^ (Ar^Dipp2^=C_6_H_3_‐2,6‐(C_6_H_3_‐2,6‐^*i*^Pr_2_)_2_) to precursor **1** generates as well compound **3** along with the chloride‐bridged digold species [(PMe_2_Ar^Dipp2^)_2_Au_2_(*μ*‐Cl)]. This contrasts with the aforementioned inability of silver salts to abstract the chloride substituent. Besides, we found slight NMR spectroscopic differences for the products derived from reactions of **1** with AgNTf_2_ versus [CPh_3_][B(C_6_F_5_)_4_] and those with NaBAr^F^ versus (PMe_2_Ar^Dipp2^)Au(NTf_2_) that we attribute to counter‐anion effects (see Figure S1 in Supporting Information).

It seems clear that the formation of a transient cationic germanium site is central for the facile activation of a benzylic C−H bond that leads to **3**. As such, this bond activation does not take place from **1** even under harsh conditions (100 °C, 48 h). In fact, germylenes are highly reluctant to insertion into C−H bonds^[18]^ and the only other example of terphenyl benzylic C−H bond activation in a tetrylene relied on the use of an extremely σ‐donating boryl ligand,^[19]^ while herein the cooperative participation of both Ge and Rh sites is crucial. The mechanism by which compound **3** is formed has been investigated and shall be discussed along these lines.

**Figure 2:**
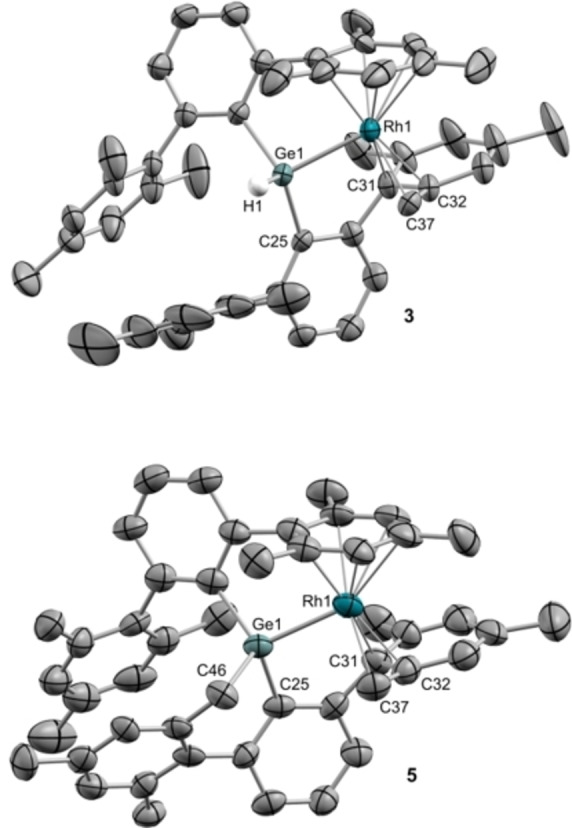
ORTEP diagrams of 3 and 5. Counter‐anions, solvent molecules and most hydrogen atoms are excluded for clarity, and thermal ellipsoids are set at 50 % probability.

Compounds **2** and **3** were unequivocally characterized by X‐ray diffraction as pseudoallylic species (Figure 2 and Figure S2), with *η* ^6^‐coordination to the alternate terphenyl substituent being retained. Pseudoallylic coordination is defined by average Rh−C bond distances of 2.28 (CH_2_), 2.13 (C_ortho_) and 2.18 Å (C_ipso_), while other geometric parameters are comparable to those of **1**.

**Scheme 3:**
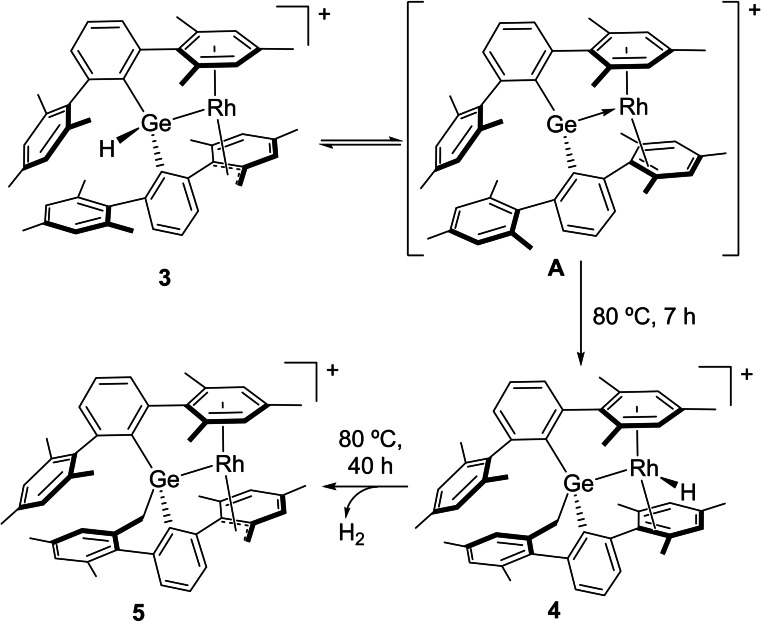
Formation of compounds **4** and **5** by stepwise double hydride/methylene migration followed by dehydrogenative C−H bond activation.

Heating compound **3** in benzene or toluene solution for seven hours results in its quantitative conversion into **4**, characterized by a distinctive low‐frequency ^1^H NMR signal at −18.12 ppm (^1^ *J*_HRh_=21.6 Hz) due to a hydride ligand bound to rhodium (Scheme 3). A thorough analysis of multinuclear mono and bidimensional NMR spectra reveals that only one of the benzylic positions of the mesityl rings is activated, although it has formally migrated to the germanium center. This formal double hydride/methylene migration between germanium and rhodium represents a rare example of the potential for cooperation in this type of hybrid main group/transition metal complex. As further investigated by computational means (see below), it must be noted that the alleged migration implies the activation of another methyl group from a different xylyl ring. Thus, formation of **4** most likely implies accessing a reactive germylene intermediate as **A** in Scheme 3, in turn the parent species from which the initial C−H activation to yield **3** after chloride abstraction would take place.

The appearance of **4** was accompanied by minute amounts (<5 %) of another species (**5**) for which the hydride ligand vanishes from the ^1^H NMR spectrum. Further heating for 40 h results in clean and complete conversion into **5**. This compound conserves the AB system observed by ^1^H NMR in complex **4**, with two signals at 2.50 (d, ^3^ *J*_HRh_=14.4 Hz) and 2.06 ppm, while also incorporates a related AB pattern (broad signals at 3.37 and 0.54 ppm) analogous to that found in compounds **2** and **3**. This suggests that two benzylic positions have now been activated with concomitant release of dihydrogen. This is in accordance with a total number of ten resonances accounting for three protons each between 1.24 and 2.21 ppm due to the methyl groups. This assumption was corroborated by X‐ray diffraction studies (Figure 2), where one of the terphenyl substituents is doubly cyclometalated to both germanium and rhodium in *η* ^1^ and *η* ^3^‐fashion, respectively, a transformation that we believe finds no precedent in the chemistry of tetrylenes. The dehydrogenation event is not reversible upon exposure to H_2_ atmosphere (2 atm). In fact, no deuterium incorporation at the benzylic positions is observed upon heating compound **5** with D_2_ (50 °C, 8 h). Exchange spectroscopy (EXSY) experiments did not allowed us to identify any chemical exchange processes in **5**, and the same results were obtained for compounds **3** and **4**. For the former, exposure to D_2_ did not reveal any isotopic labelling either.

**Figure 3:**
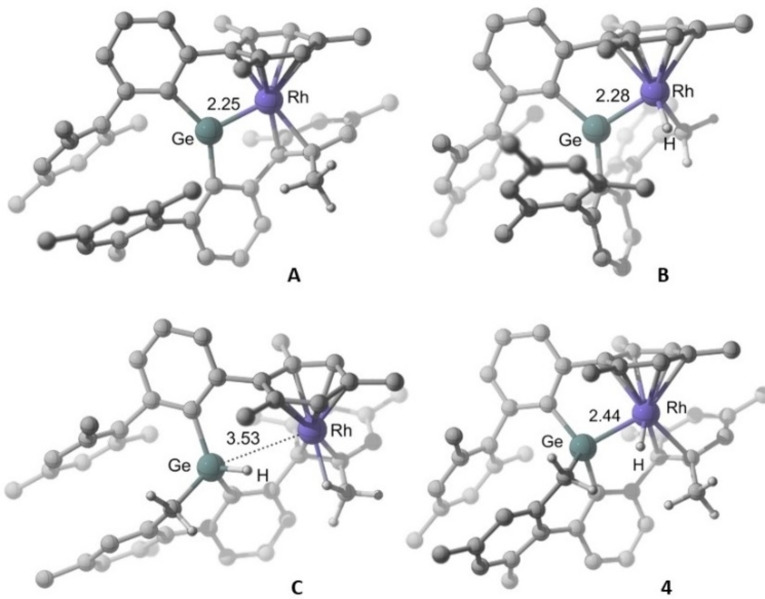
DFT optimized geometries of relevant intermediates. Ge−Rh distances are in Å. Most hydrogen atoms have been omitted for clarity. The dotted line in C does not indicate a Ge−Rh interaction.

Mechanistic understanding on this dehydrogenative double C−H bond activation is central to further explore the cooperative potential of this kind of tetrylene‐transition metal systems. Investigation of the formation of species **3**–**5** by DFT methods (SMD‐ωB97XD/6‐31g(d,p)/SDD level; see the Supporting Information for details) were carried out starting from the cationic germylene‐rhodium **A** that results from elimination of the chloride atom in **1** (Figure 3). **A** retains the coordination environment around rhodium, whereas the expected trigonal planar geometry is found around the germanium with a Ge−Rh distance of 2.25 Å.

**Figure 4:**
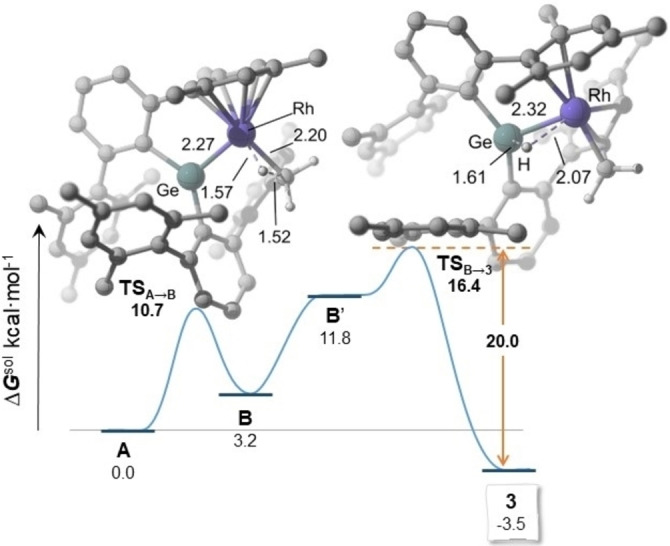
DFT‐calculated energy profile from **A** to **3** and optimized geometries for the transition states (distances in Å, most hydrogen atoms have been omitted for clarity). Dashed lines indicate bonds being broken and formed.

The calculations favor initial C−H activation at rhodium,^[20]^ which occurs through a low energy barrier (Δ*G* from **A**) of ca. 10 kcal ⋅ mol^−1^, to afford a new germylene intermediate, **B**, only 3.2 kcal ⋅ mol^−1^ higher in energy than **A** and featuring a formally Rh(III) hydride moiety (Figure 4). Despite the change in formal oxidation state of the rhodium, the Ge−Rh distance increases only slightly by 0.03 Å. Thus, this metrics does not constitute a clear indicator for changes in the Ge−Rh interaction, which was probed instead by Quantum Theory of Atoms in Molecules (AIM) and Natural Bonding Orbital (NBO) analyses as discussed below. Species **3** would in turn result from hydride migration from Rh to Ge. This transformation requires a change in the coordination mode of the aryl moiety initially coordinated as *η* ^6^ in **B**. Thus, isomer **B′**, features a shorter Rh*H*⋅⋅⋅Ge interaction (2.48 Å vs. 2.61 Å in **B**) and longer rhodium arene distances (Figure S4), as a result of which it lies 8.6 kcal ⋅ mol^−1^ above **B** in the energy profile. **B′** is directly connected to **3** through **TS_B′→3_**, being the energy barrier of 4.6 kcal ⋅ mol^−1^. The overall transformation from **A** is exergonic by 3.5 kcal ⋅ mol^−1^ and has an overall barrier of ca. 16 kcal mol^−1^, which results in a reverse barrier of 20 kcal mol^−1^.^[21]^

**Figure 5:**
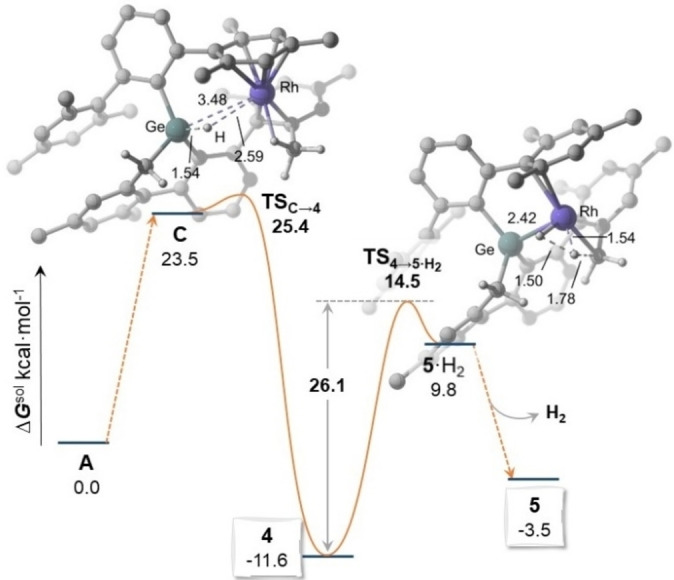
DFT‐calculated energy profile from **A** to **5** and optimized geometries for the transition states (distances in Å, most hydrogen atoms have been omitted for clarity). Dashed lines indicate bonds being broken and formed.

Formation of **4** and **5** from **3** under forcing conditions implies accessing the reacting germylene intermediate **A**, and may involve the addition product, the germyl **C**. The DFT‐optimized geometry of **C** (Figure 3) exhibits a Ge−Rh distance of 3.53 Å, precluding significant interaction between the two atoms, as confirmed by NBO analysis (Wiberg bond index=0.02 *v*. 0.54 in **3** and 0.90 in **A**). Notwithstanding, QTAIM analysis of the electron density hints at a weak interaction between the GeH fragment and the rhodium (Ge*H*⋅⋅⋅Rh distance=2.65 Å) as it locates a bond critical point (bcp) and bond path between the hydrogen and rhodium atoms (Figure S10). The rhodium in **C** is partly stabilized by the establishment of an additional agostic interaction with one methyl from the metalated terphenyl. However, this species is, as expected,^[18]^ a high energy intermediate, located 23.5 kcal ⋅ mol^−1^ above **A** (Figure 5), which evolves almost without a barrier through **TS_C→4_** (Δ*G* ^≠^=25.4 kcal ⋅ mol^−1^ from **A**) to a stable rhodium hydride species that maps onto the experimentally detected **4** (Δ*G* ^0^=−11.6 kcal ⋅ mol^−1^ from **A**). **TS_C→4_** retains several structural characteristics of **C** including long Ge−Rh and Ge*H*⋅⋅⋅Rh distances (3.48 Å, and 2.59 Å respectively), which suggests that it is in a flat region of the potential energy surface. Hydride **4** therefore results from H migration from Ge to Rh with reforming of the Ge−Rh bond (2.44 Å). It is pertinent to say at this point that transition states connecting **A** and **B** with **4** were also located. These steps imply C−H activation across the Ge−Rh linkage and have barriers of 33.6 and 34.3 kcal ⋅ mol^−1^ respectively, which is at odds with the isolation of **4** as shown next. Thus, formation of germyl **5** requires H_2_ elimination, which in turn implies a further C−H activation event through **TS_4→5 ⋅ H2_**. The calculated barrier of 26.1 kcal ⋅ mol^−1^ is higher than that for the formation of **4** from **A**, but lower than those for C−H activation across the Ge−Rh bonds of **A** and **B. TS_4→5 ⋅ H2_** has structural characteristics of a rhodium dihydride species with Rh−H distances of 1.53 and 1.54 Å and the H−H distance is 1.50 Å. **TS_4→5 ⋅ H2_** yields an unstable Rh dihydride species, **5 ⋅ H_2_** (▵*G*°=9.8 kcal mol^−1^ from **A**, Figure S5) from which exergonic H_2_ elimination readily affords **5**.

**Figure 6:**
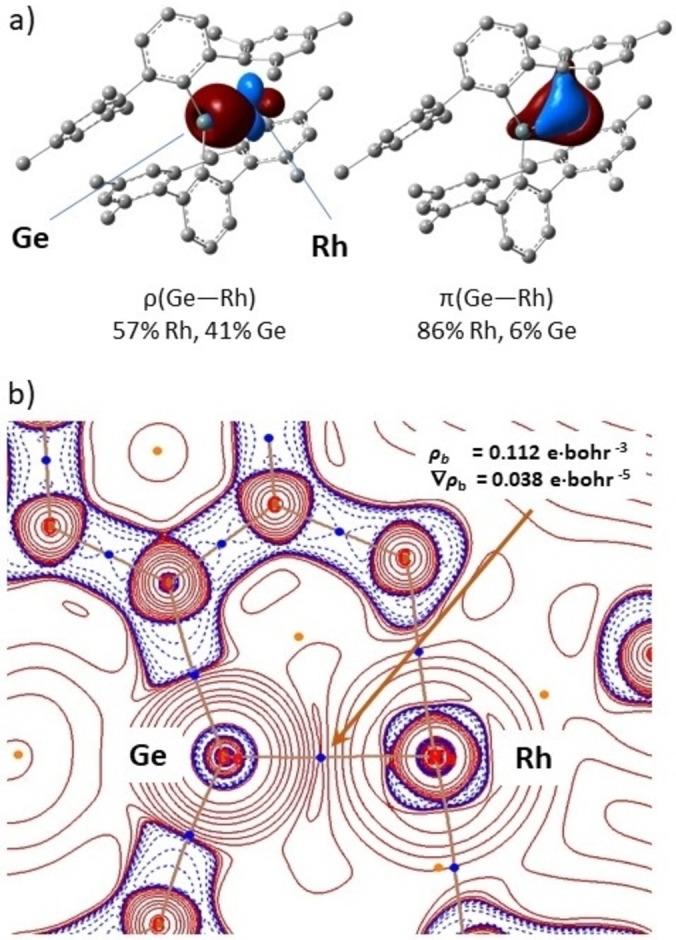
a) NLMOs (0.05 isovalue) for the main contributions to the Ge−Rh bonding in **A**. b) QTAIM analysis of **A**: Bond critical points of the electron density, ***ρ***, and bond paths superimposed on the Laplacian, ∇^2^ ***ρ*** in one C_Aryl_‐Ge−Rh plane (solid and dashed lines are for positive and negative values).

We have used QTAIM and NBO analyses (SMD‐ωB97XD/Def2TZVP level) to probe the germanium‐rhodium interaction throughout the reaction pathway. Focusing on the formation of **3** from **A**, QTAIM analysis reveals electron densities at the Ge−Rh bcps, *ρ*_b_, in the range 0.112–0.083 e ⋅ bohr^−3^, being greater for the germylenes **A**, **B′** and for **TS_B′→3_** than for germyl **3** (Figure 6b) and Table S2). The values of the Laplacian of the electron density at these bcps (∇^2^ *ρ*_b_) are close to zero for all species considered, but it is negative for germyl **3** only. Consistent with this, the |*V*_b_|/*G*_b_ ratio (between the absolute electronic potential energy and kinetic energy densities) is between 1 and 2 for germylenes **A** and **B′** (and **TS_B′→3_**), and greater than 2 for germyl **3**. These results indicate a high degree of covalency for the Ge−Rh bond, but only in the case of **3** the data agrees with a classical covalent interaction.^[22]^ Additionally, the ellipticity, *ϵ*_b_, of the Ge−Rh bonds is largest in germylene **A** at 0.191, whereas germyl **3** exhibits the smallest value at 0.033, consistent with the Ge−Rh bond in germylenes, particularly **A**, having greater double bond character. NBO analysis of the same species depicts the Lewis like electronic structure of **A** with a Natural Localized Molecular Orbital (NLMO) between the germanium and rhodium atoms having 57 % rhodium and 40 % germanium character for the σ component of the bond (Rh/Ge d/sp^2^) and two NLMOs for the π contribution with 8.0 and 2.4 % germanium character (Rh/Ge d/p; Figure 6a). NLMOs can be thought of as doubly‐occupied localized orbitals derived from the admixture of their parent donor‐NBOs and orbitals from the molecular fragments onto which there are delocalized. In **B′** the rhodium contribution to the σ component (Rh/Ge d/sp^2^) has 51 % and 43 % Rh/Ge character, and the Ge contribution to the π components decrease to 3.6 and 1.2 %. Less mixing of germanium orbitals in the π components of the Ge−Rh bond indicate less back donation from rhodium. In germyl **3**, a very different bonding scheme is depicted, consistent with a single bond character of the Ge−Rh bond: only one significant component to the bond, constructed from a d filled orbital on rhodium and one empty p orbital on the germanium results in a NLMO having 71 % and 25 % Rh/Ge character. These findings illustrate how the Ge−Rh linkage responds to the rearrangements from germylene to germyl experienced by the former, reinforcing the notion of assistance of the latter in the reactivity of the complex.

## Conclusion

In summary, the reactivity studies described in the foregoing sections constitute a remarkable illustration of the cooperative potential of transition metal tetrylenes. Chloride abstraction from a rhodium‐embraced germyl compound initiates a highly unusual series of bond breaking events that result in the double C−H bond activation of two benzylic positions with concomitant release of dihydrogen. Computational studies provide a mechanistic picture for the above transformation, emphasizing the key cooperative action of rhodium and germanium. The process involves up to three C−H bond cleavage steps, as well as reversible hydride migration and formal hydrocarbyl migration between germanium and rhodium. These observations highlight the prospects for cooperative bond activation and catalysis of transition metal tetrylenes, which may surpass the more widely explored base‐stabilized tetrylenes as ligands for transition metals.

## Supporting Information

Synthesis, characterization, NMR spectra and analysis, and computational details can all be found in the Supporting Information.

Deposition Numbers 2083762 (for **1**), 2083760 (for **2**), 2083759 (for **3**), and 2083761 (for **5**) contain the supplementary crystallographic data for this paper. These data are provided free of charge by the joint Cambridge Crystallographic Data Centre and Fachinformationszentrum Karlsruhe Access Structures service www.ccdc.cam.ac.uk/structures.

## Conflict of interest

The authors declare no conflict of interest.

## Supporting information

As a service to our authors and readers, this journal provides supporting information supplied by the authors. Such materials are peer reviewed and may be re‐organized for online delivery, but are not copy‐edited or typeset. Technical support issues arising from supporting information (other than missing files) should be addressed to the authors.

Supporting Information
