# Supplementary material for: Dehydrogenative Double C−H Bond Activation in a Germylene‐Rhodium Complex
Source: Chemistry. 2021 Oct 27;27(66):16422–8. doi: 10.1002/chem.202102529 (PMC9297988; doi:10.1002/chem.202102529)
Supplement: Supplementary file 1 — Supporting Information [file CHEM-27-16422-s001.pdf]

Chemistry–A European Journal

Supporting Information

Dehydrogenative Double C–H Bond Activation in a Germylene-Rhodium Complex

Sonia Bajo, María M. Alcaide, Joaquín López-Serrano, and Jesús Campos*

1. Synthesis and characterization of new compounds S2

2. Solution NMR analysis of compounds of type **2** and **3** S10

3. X-Ray structural characterization of new compounds. S11

4. Computational details S14

5. NMR spectra of new compounds S22

6. References S29

1. Synthesis and characterization of new compounds

General considerations. All manipulations were carried out using standard Schlenk and glove-box techniques, under an atmosphere of argon and of high purity nitrogen, respectively. All solvents were dried, stored over 4 Å molecular sieves, and degassed prior to use. *N*-pentane (C_5H_{12}) were distilled under nitrogen over sodium. [D6]Benzene was distilled under argon over sodium/benzophenone. Compounds [$(Ar^{Mes2})_2$Ge:] (Ar^{Mes} = C_6H_3-2,6-(C_6H_2-2,4,6-$Me_3)_2$)[1], [RhCl(COD)]$_2$ (COD = 1,5-ciclooctadinene)[2] and NaBAr$_F$[3] were prepared as described previously. Other chemicals were commercially available and used as received. Solution NMR spectra were recorded on Bruker AMX-300, DRX-400 and DRX-500 spectrometers. Spectra were referenced to external SiMe4 (δ: 0 ppm) using the residual proton solvent peaks as internal standards (^{1}H NMR experiments), or the characteristic resonances of the solvent nuclei (^{13}C NMR experiments), while ^{31}P was referenced to H_3PO_4. Spectral assignments were made by routine one- and two-dimensional NMR experiments (^{1}H, ^{13}C{^{1}H}, ^{31}P{^{1}H}, COSY, NOESY, HSQC and HMBC) where appropriate. Spectroscopic NMR resonances due to BAr^F anion: ^{1}H: δ 8.37 (s, 8H, *o*-Ar) and 7.67 (s, 4H, *p*-Ar) and ^{13}C: 162.3 (q, $^1J_{CB}$ = 50 Hz, *ipso*-Ar), 135.1 (s, *o*-Ar) and 117.7 (s, *p*-Ar). For elemental analyses a LECO TruSpec CHN elementary analyzer, was utilized.

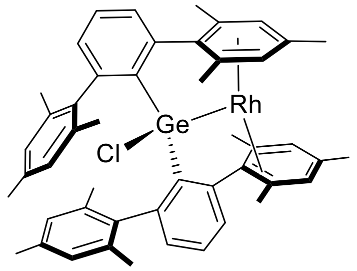

Compound 1. A solution of [(Ar^{Mes2})$_2$Ge:] (300 mg, 0.43 mmol) and [RhCl(COD)]$_2$ (COD = 1,5-Cyclooctadiene) (106 mg, 0.21 mmol) in 5 mL of toluene was heated at 80 °C in a Schlenk flask equipped with a Teflon stopcock overnight, and a red-brown solution was formed. After this time, it was allowed to reach room temperature and the solvent was removed in vacuo. The resulting solid was washed with n-pentane and dried under vacuum to afford a dark orange solid. (310 mg, 86 %).

Anal. Calcd for $C_{48}H_{50}ClGeRh$: C, 68.8; H, 6.0. Found: C, 68.9; H, 6.2.

^{1}H NMR (400 MHz, C_6D_6, 298 K): δ 7.10 (t, $^3J_{HH}$ = 7.3 Hz, 2H, CH), 6.96 (s, 2H, CH), 6.85 (d, $^3J_{HH}$ = 7.3 Hz, 2H, CH), 6.79 (s, 2H, CH), 6.75 (d, $^3J_{HH}$ = 7.3 Hz, 2H, CH), 6.64 (s, 2H, CH), 5.16 (s, 2H, CH), 2.49 (s, 6H, CH_3 Ar^{Mes}), 2.29 (s, 6H, CH_3 Ar^{Mes}), 2.11 (s, 6H, CH_3 Ar^{Mes}), 2.04 (s, 6H, CH_3 Ar^{Mes}), 1.99 (s, 6H, CH_3 Ar^{Mes}), 1.91 (s, 6H, CH_3 Ar^{Mes}).

^{13}C{^{1}H} NMR (100.63 MHz, C_6D_6, 298 K): δ 152.3 (d, $^2J_{CRh}$ = 7 Hz, *i*-C_6H_3), 147.1 (s, Cq), 146.1 (s, Cq), 140.7 (s, Cq), 137.0 (s, Cq), 135.4 (s, Cq), 135.0 (s, Cq), 131.3 (s, CH), 128.9 (s, *p*-C_6H_3), 128.3 (s, CH), 126.3 (s, Cq), 119.9 (d, J_{CRh} = 9 Hz, CH), 100.0 (d, J_{CRh} = 11 Hz, Cq), 97.0 (d, J_{CRh} = 8 Hz, Cq), 23.5 (s, *C*H_3 Ar^{Mes}), 22.0 (s, *C*H_3 Ar^{Mes}), 21.8 (s, *C*H_3 Ar^{Mes}), 21.1 (s, *C*H_3 Ar^{Mes}), 21.0 (s, *C*H_3 Ar^{Mes}), 20.9 (s, *C*H_3 Ar^{Mes}).

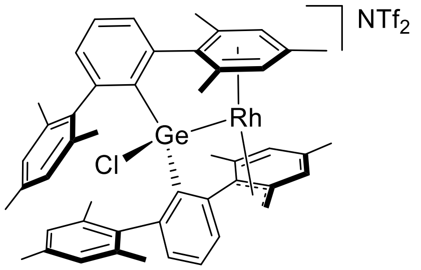

Compound 2-NTf$_2$. In a NMR tube, to a solution of compound **1** (30 mg, 0.036 mmol) in C_6D_6 (0.5 mL) was added silver triflimide (14 mg, 0.036 mmol). The orange solution became yellow after 5 min. Compound **2-NTf$_2$** can be crystallized by slow evaporation of the C_6D_6 solution (27 mg, 67%).

Anal. Calcd for $C_{50}H_{49}ClF_6GeNO_4RhS_2$: C, 53.8; H, 4.4. Found: C, 54.0; H, 4.4.

^{1}H NMR (400 MHz, C_6D_6, 298 K): δ 7.12 (m, 1H, CH), 7.06 (t, $^3J_{HH}$ = 7.5 Hz, 1H, CH), 6.89 (s, 1H, CH), 6.85 (s, 1H, CH), 6.83 (s, 1H, CH), 6.72 (s, 1H, CH), 6.63 (overlapping signals, 3H, CH), 6.49 (overlapping signals, 2H, CH), 6.18 (s, 1H, CH), 5.90 (d, $^3J_{HH}$ = 7.5 Hz, 1H, CH), 5.06 (s, 1H, CH), 3.93 (br, 1H, Ar-C*H*$_2$Rh), 2.47 (s, 3H, CH$_3$ Ar^{Mes}), 2.32 (s, 3H, CH$_3$ Ar^{Mes}), 2.28 (s, 3H, CH$_3$ Ar^{Mes}), 2.00 (s, 3H, CH$_3$ Ar^{Mes}), 1.99 (s, 3H, CH$_3$ Ar^{Mes}), 1.98 (s, 3H, CH$_3$ Ar^{Mes}), 1.88 (s, 3H, CH$_3$ Ar^{Mes}), 1.83 (s, 3H, CH$_3$ Ar^{Mes}), 1.75 (s, 3H, CH$_3$ Ar^{Mes}), 1.65 (s, 3H, CH$_3$ Ar^{Mes}), 1.36 (br, 1H, Ar-C*H*$_2$Rh), 1.27 (s, 3H, CH$_3$ Ar^{Mes}).

^{13}C{^{1}H} NMR (100.63 MHz, C_6D_6, 298 K): δ 156.2 (s, Cq), 147.0 (s, Cq), 146.9 (s, Cq), 142.8 (s, Cq), 140.6 (d, J_{CRh} = 5 Hz, Cq), 139.1 (s, Cq), 137.3 (s, Cq), 137.2 (s, Cq), 137.1 (s, Cq), 136.8 (s, Cq), 136.7 (s, Cq), 136.1 (s, Cq), 135.7 (s, Cq), 135.6 (s, Cq), 133.5 (s, CH), 132.4 (s, CH), 131.2 (s, CH),130.2 (s, CH), 130.1 (s, CH), 129.7 (s, CH), 128.9 (s, CH), 128.5 (s, CH), 126.7 (s, CH), 124.3 (s, CH), 123.0 (d, J_{CRh} = 5 Hz, Cq), 121.8 (s, CH), 121.6 (s, Cq), 120.2 (s, Cq), 118.4 (s, Cq), 116.1 (d, J_{CRh} = 4 Hz, Cq), 104.4 (s, CH), 101.7 (d, J_{CRh} = 5 Hz, Cq), 94.4 (d, J_{CRh} = 13 Hz, Cq), 42.3 (d, J_{CRh} = 12 Hz, Ar-*C*H$_2$Rh), 22.2 (s, *C*H$_3$ Ar^{Mes}), 21.8 (s, *C*H$_3$ Ar^{Mes}), 21.7 (s, *C*H$_3$ Ar^{Mes}), 21.4 (s, *C*H$_3$ Ar^{Mes}), 21.0 (s, *C*H$_3$ Ar^{Mes}), 20.8 (s, *C*H$_3$ Ar^{Mes}), 20.7 (s, *C*H$_3$ Ar^{Mes}), 20.6 (s, *C*H$_3$ Ar^{Mes}), 19.0 (s, *C*H$_3$ Ar^{Mes}), 18.2 (s, *C*H$_3$ Ar^{Mes}), 17.1 (s, *C*H$_3$ Ar^{Mes}).

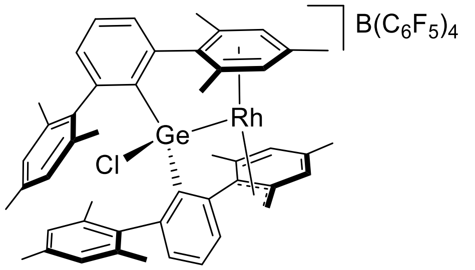

Compound 2. In a NMR tube, to a solution of compound **1** (30 mg, 0.036 mmol) in C_6D_6 (0.5 mL) was added trityl tetrakis(pentafluorophenyl)borate (33 mg, 0.036 mmol). The orange solution became yellow after 5 min. A mixture of **2** and $CHPh_3$ was obtained.

Signals corresponding to **2** are the following:

^{1}H NMR (400 MHz, C_6D_6, 298 K): δ 7.02 (m, 1H, CH), 6.91 (d, $^3J_{HH}$ = 7.6 Hz, 1H, CH), 6.82 (s, 1H, CH), 6.73 (s, 1H, CH), 6.64 (overlapping signals, 4H, CH), 6.56 (s, 1H, CH), 6.51 (t, $^3J_{HH}$ = 7.6 Hz, 2H, CH), 5.86 (d, $^3J_{HH}$ = 7.8 Hz, 1H, CH), 5.62 (s, 1H, CH), 4.23 (s, 1H, CH), 3.62 (br, 1H, Ar-C*H*$_2$Rh), 2.33 (s, 3H, CH_3 Ar^{Mes}), 2.28 (s, 3H, CH_3 Ar^{Mes}), 2.22 (s, 3H, CH_3 Ar^{Mes}), 1.90 (s, 3H, CH_3 Ar^{Mes}), 1.86 (s, 3H, CH_3 Ar^{Mes}), 1.83 (s, 6H, CH_3 Ar^{Mes}), 1.64 (s, 3H, CH_3 Ar^{Mes}), 1.48 (s, 3H, CH_3 Ar^{Mes}), 1.42 (s, 3H, CH_3 Ar^{Mes}), 1.31 (br, 1H, Ar-C*H*$_2$Rh), 1.25 (s, 3H, CH_3 Ar^{Mes}).

^{13}C{^{1}H} NMR (100.63 MHz, C_6D_6, 298 K): δ 155.8 (s, Cq), 147.1 (s, Cq), 142.5 (s, Cq), 142.0 (s, Cq), 140.2 (d, J_{CRh} = 5 Hz, Cq), 138.6 (s, Cq), 137.4 (s, Cq), 137.0 (s, Cq), 136.6 (s, Cq), 136.4 (s, Cq), 135.7 (s, Cq), 135.4 (s, Cq), 134.1 (s, CH), 132.8 (s, CH), 131.3 (s, CH), 130.5 (s, CH), 130.2 (s, CH), 130.0 (s, CH), 128.6 (s, CH), 126.7 (s, Cq), 126.0 (s, Cq), 123.2 (s, CH), 121.5 (s, CH), 119.4 (s, Cq), 116.7 (d, J_{CRh} = 5 Hz, Cq), 103.4 (s, CH), 102.0 (d, J_{CRh} = 5 Hz, Cq), 94.8 (d, J_{CRh} = 13 Hz, Cq), 42.7 (d, J_{CRh} = 12 Hz, Ar-*C*H$_2$Rh), 22.1 (s, *C*H$_3$ Ar^{Mes}), 21.7 (s, *C*H$_3$ Ar^{Mes}), 21.6 (s, *C*H$_3$ Ar^{Mes}), 21.1 (s, *C*H$_3$ Ar^{Mes}), 21.0 (s, *C*H$_3$ Ar^{Mes}), 20.8 (s, *C*H$_3$ Ar^{Mes}), 20.6 (s, *C*H$_3$ Ar^{Mes}), 20.4 (s, *C*H$_3$ Ar^{Mes}), 18.9 (s, *C*H$_3$ Ar^{Mes}), 17.4 (s, *C*H$_3$ Ar^{Mes}), 16.5 (s, *C*H$_3$ Ar^{Mes}).

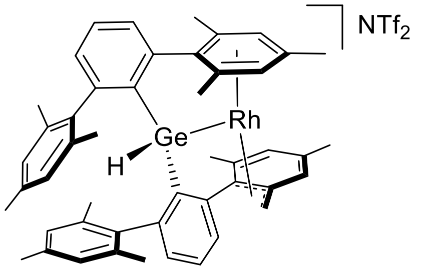

Compound 3-NTf$_2$. In a NMR tube, to a solution of compound **1** (20 mg, 0.024 mmol) in C_6D_6 (0.5 mL) was added [PMe$_2$Ar^{Dipp_2}]AuNTf$_2$ (45 mg, 0.048 mmol). The orange solution became yellow after 5 min. A mixture of **3-NTf$_2$** and [(PMe$_2$ArDipp$_2$)$_2$Au$_2$(μ-Cl)][NTf$_2$] was obtained. Compound **3-NTf$_2$** could be crystallized by slow evaporation of the C_6D_6 solution.

Signals corresponding to **3-NTf$_2$** are the following:

^{1}H NMR (400 MHz, C_6D_6, 298 K): δ 7.00 (d, $^3J_{HH}$ = 7.6 Hz, 1H, CH), 6.93 (overlapping signals, 4H, CH), 6.76 (s, 1H, CH), 6.70 (s, 1H, CH), 6.64 (s, 1H, CH), 6.60 (overlapping signals, 2H, CH), 6.43 (d, $^3J_{HH}$ = 7.6 Hz, 1H, CH), 6.34 (s, 1H, CH), 5.96 (s, 1H, Ge-*H*), 5.86 (t, $^3J_{HH}$ = 4.5 Hz, 1H, CH), 5.08 (s, 1H, CH), 3.85 (br, 1H, Ar-C*H*$_2$Rh), 2.36 (s, 3H, CH$_3$ Ar^{Mes}), 2.31 (s, 3H, CH$_3$ Ar^{Mes}), 2.26 (s, 3H, CH$_3$ Ar^{Mes}), 2.20 (s, 3H, CH$_3$ Ar^{Mes}), 2.00 (s, 3H, CH$_3$ Ar^{Mes}), 1.87 (s, 3H, CH$_3$ Ar^{Mes}), 1.83 (s, 3H, CH$_3$ Ar^{Mes}), 1.70 (s, 3H, CH$_3$ Ar^{Mes}), 1.66 (s, 3H, CH$_3$ Ar^{Mes}), 1.57 (s, 3H, CH$_3$ Ar^{Mes}), 1.45 (s, 3H, CH$_3$ Ar^{Mes}), 1.17 (br, 1H, Ar-C*H*$_2$Rh).

^{13}C{^{1}H} NMR (100.63 MHz, C_6D_6, 298 K): δ 151.2 (s, Cq), 148.9 (s, Cq), 148.1 (s, Cq), 145.9 (s, Cq), 145.5 (d, J_{CRh} = 11 Hz, Cq), 145.4 (s, Cq), 141.9 (s, Cq), 139.1 (s, Cq), 137.2 (s, Cq), 136.0 (s, Cq), 135.9 (s, Cq), 135.6 (s, Cq), 134.8 (s, Cq), 134.2 (s, Cq), 132.2 (s, CH), 130.4 (s, CH), 129.5 (s, CH), 129.3 (s, CH), 129.1 (s, CH), 129.0 (s, CH), 128.6 (s, CH), 126.0 (s, Cq), 124.7 (s, CH), 122.4 (s, Cq), 121.0 (s, Cq), 119.2 (d, J_{CRh} = 19 Hz, Cq), 110.9 (d, J_{CRh} = 4 Hz, Cq), 101.9 (d, J_{CRh} = 5 Hz, Cq), 101.1 (s, CH), 94.6 (d, J_{CRh} = 13 Hz, Cq), 40.2 (d, J_{CRh} = 12 Hz, Ar-*C*H$_2$Rh), 22.3 (s, *C*H$_3$ Ar^{Mes}), 21.6 (s, *C*H$_3$ Ar^{Mes}), 21.3 (s, *C*H$_3$ Ar^{Mes}), 21.2 (s, *C*H$_3$ Ar^{Mes}), 21.1 (s, *C*H$_3$ Ar^{Mes}), 20.8 (s, *C*H$_3$ Ar^{Mes}), 20.4 (s, *C*H$_3$ Ar^{Mes}), 19.6 (s, *C*H$_3$ Ar^{Mes}), 18.3 (s, *C*H$_3$ Ar^{Mes}), 18.0 (s, *C*H$_3$ Ar^{Mes}), 15.8 (s, *C*H$_3$ Ar^{Mes}).

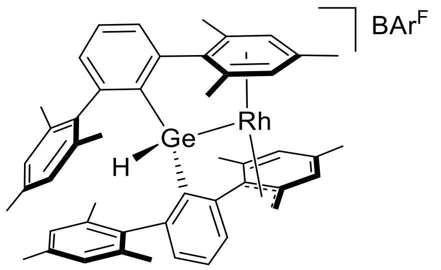

Compound 3. To a solution of compound **1** (50 mg, 0.06 mmol) in 3 mL of toluene was added sodium tetrakis[3,5-bis(trifluoromethyl)phenyl]borate (53 mg, 0.06 mmol) in a Schlenk flask equipped with a Teflon stopcock. The orange solution became yellow after 12 hours at room temperature. After this time, the resulting suspension was filtered through celite and the solvent was removed in vacuo. The resulting solid was washed with n-pentane and dried under vacuum to afford a yellow solid (76 mg, 76%).

Anal. Calcd for $C_{80}H_{62}BF_{24}GeRh$: C, 57.7; H, 3.8. Found: C, 57.8; H, 3.7.

^{1}H NMR (400 MHz, C_6D_6, 298 K): δ 8.49 (s, 8H, *o*-Ar), 7.80 (s, 4H, *p*-Ar), 7.08 (t, $^3J_{HH}$ = 7.6 Hz, 1H, CH), 6.86 (s, 1H, CH), 6.83 (d, $^3J_{HH}$ = 7.6 Hz, 1H, CH), 6.73 (overlapping signals, 6H, CH), 6.64 (s, 1H, CH), 6.57 (d, $^3J_{HH}$ = 7.6 Hz, 1H, CH), 6.02 (s, 1H, Ge-*H*), 5.95 (d, $^3J_{HH}$ = 7.6 Hz, 1H, CH), 5.45 (s, 1H, CH), 4.22 (s, 1H, CH), 3.44 (br, 1H, Ar-CH_2Rh), 2.43 (s, 3H, CH_3 Ar^{Mes}), 2.40 (s, 3H, CH_3 Ar^{Mes}), 2.15 (s, 3H, CH_3 Ar^{Mes}), 1.90 (s, 6H, CH_3 Ar^{Mes}), 1.79 (s, 3H, CH_3 Ar^{Mes}), 1.76 (s, 3H, CH_3 Ar^{Mes}), 1.66 (s, 3H, CH_3 Ar^{Mes}), 1.54 (s, 3H, CH_3 Ar^{Mes}), 1.51 (s, 3H, CH_3 Ar^{Mes}), 1.30 (s, 3H, CH_3 Ar^{Mes}), 1.17 (br, 1H, Ar-CH_2Rh).

^{13}C{^{1}H} NMR (100.63 MHz, C_6D_6, 298 K): δ 162.3 (q, $^1J_{CB}$ = 50 Hz, *ipso*-Ar), 150.7 (s, Cq), 148.9 (s, Cq), 146.6 (s, Cq), 145.4 (s, Cq), 144.3 (s, Cq), 141.6 (s, Cq), 138.3 (d, J_{CRh} = 11 Hz, Cq), 137.8 (s, Cq), 136.6 (s, Cq), 136.4 (s, Cq), 136.1 (s, Cq), 135.1 (s, *o*-Ar), 133.5 (s, Cq), 133.0 (s, CH), 130.9 (s, Cq), 130.0 (s, CH), 129.8 (s, CH), 129.4 (s, CH), 129.1 (s, Cq), 128.9 (s, CH), 126.2 (s, Cq), 125.8 (s, CH), 123.5 (s, CH), 123.2 (s, Cq), 121.9 (s, CH), 121.1 (s, Cq), 120.8 (s, Cq), 118.4 (s, Cq), 117.7 (s, *p*-Ar), 111.3 (s, Cq), 102.4 (s, Cq), 99.5 (s, CH), 95.6 (d, J_{CRh} = 12 Hz, CH), 40.4 (d, J_{CRh} = 13 Hz, Ar-CH$_2$Rh), 22.2 (s, CH_3 Ar^{Mes}), 21.5 (s, CH_3 Ar^{Mes}), 21.2 (s, CH_3 Ar^{Mes}), 20.7 (s, CH_3 Ar^{Mes}), 20.3 (s, CH_3 Ar^{Mes}), 19.4 (s, CH_3 Ar^{Mes}), 17.6 (s, CH_3 Ar^{Mes}), 17.2 (s, CH_3 Ar^{Mes}), 15.0 (s, CH_3 Ar^{Mes}).

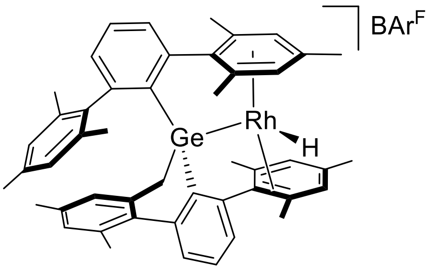

Compound 4. In a NMR tube, a solution of **3** (30 mg, 0.018 mmol) in C_6D_6 (0.5 mL) was heated at 80 °C during 7 hours. After this time, it was allowed to reach room temperature and the solvent was removed in vacuo. The resulting solid was washed with n-pentane and dried under vacuum to afford an orange solid. (16 mg, 53 %).

Anal. Calcd for $C_{80}H_{62}BF_{24}GeRh$: C, 57.7; H, 3.8. Found: C, 57.9; H, 4.0.

^{1}H NMR (400 MHz, C_6D_6, 298 K): δ 8.37 (s, 8H, *o*-Ar), 7.67 (s, 4H, *p*-Ar), 7.03 (t, $^3J_{HH}$ = 7.7 Hz, 1H, CH), 6.99 (d, $^3J_{HH}$ = 7.7 Hz, 1H, CH), 6.95 (s, 1H, CH), 6.78 (t, $^3J_{HH}$ = 7.7 Hz, 1H, CH), 6.71 (d, $^3J_{HH}$ = 7.7 Hz, 1H, CH), 6.61 (overlapping signals, 2H, CH), 6.51 (s, 1H, CH), 6.45 (s, 1H, CH), 6.27 (s, 1H, CH), 6.07 (s, 1H, CH), 5.83 (s, 1H, CH), 5.65 (d, $^3J_{HH}$ = 7.7 Hz, 1H, CH), 3.44 (s, 1H, CH), 2.47 (d, $^3J_{HRh}$ = 14.2 Hz, 1H, Ar-CH_2Ge), 2.15 (s, 3H, CH_3 Ar^{Mes}), 2.13 (s, 3H, CH_3 Ar^{Mes}), 2.10 (s, 3H, CH_3 Ar^{Mes}), 2.08 (overlapping signal, 1H, Ar-CH_2Ge), 1.96 (s, 3H, CH_3 Ar^{Mes}), 1.89 (s, 3H, CH_3 Ar^{Mes}), 1.79 (s, 3H, CH_3 Ar^{Mes}), 1.71 (s, 3H, CH_3 Ar^{Mes}), 1.66 (s, 3H, CH_3 Ar^{Mes}), 1.34 (s, 3H, CH_3 Ar^{Mes}), 1.25 (s, 3H, CH_3 Ar^{Mes}), 1.10 (s, 3H, CH_3 Ar^{Mes}), - 18.12 (d, $^1J_{HRh}$ = 21.6 Hz 1H, Rh-*H*).

^{13}C{^{1}H} NMR (100.63 MHz, C_6D_6, 298 K): δ 162.4 (q, $^1J_{CB}$ = 50 Hz, *ipso*-Ar), 151.2 (s, Cq), 148.7 (s, Cq), 145.6 (s, Cq), 144.9 (s, Cq), 141.8 (s, Cq), 138.1 (s, Cq), 136.2 (d, J_{CRh} = 10 Hz, Cq), 135.1 (s, *o*-Ar), 134.7 (d, J_{CRh} = 7 Hz, Cq), 134.0 (s, Cq), 133.5 (d, J_{CRh} = 10 Hz, CH), 133.2 (s, Cq), 132.3 (s, CH), 131.8 (s, CH), 130.4 (s, CH), 130.0 (s, CH), 129.9 (s, CH), 129.7 (s, CH), 129.5 (s, CH), 129.4 (s, CH), 128.9 (s, Cq), 126.2 (s, Cq), 125.9 (s, CH), 125.7 (s, Cq), 124.4 (s, Cq), 123.5 (s, Cq), 120.8 (s, Cq), 117.7 (s, *p*-Ar), 105.2 (s, CH), 102.7 (s, Cq), 96.0 (s, Cq), 29.9 (br, Ar-*C*H_2Ge), 27.0 (s, *C*H_3 Ar^{Mes}), 23.4 (s, *C*H_3 Ar^{Mes}), 22.9 (s, *C*H_3 Ar^{Mes}), 21.6 (s, *C*H_3 Ar^{Mes}), 21.0 (s, *C*H_3 Ar^{Mes}), 20.6 (s, *C*H_3 Ar^{Mes}), 20.5 (s, *C*H_3 Ar^{Mes}), 20.4 (s, *C*H_3 Ar^{Mes}), 18.7 (s, *C*H_3 Ar^{Mes}), 17.6 (s, *C*H_3 Ar^{Mes}), 16.0 (s, *C*H_3 Ar^{Mes}).

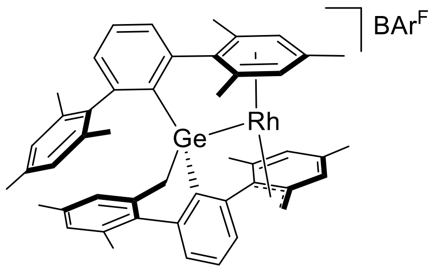

Compound 5. In a NMR tube, a solution of **3** (30 mg, 0.018 mmol) in C_6D_6 (0.5 mL) was heated at 80 °C during 48 hours. Compound **5** can be crystallized by slow evaporation of the C_6D_6 solution (23 mg, 77%).

Anal. Calcd for $C_{80}H_{60}BF_{24}GeRh$: C, 57.8; H, 3.6. Found: C, 58.0; H, 3.7.

^{1}H NMR (400 MHz, C_6D_6, 298 K): δ 8.37 (s, 8H, *o*-Ar), 7.67 (s, 4H, *p*-Ar), 7.06 (t, $^3J_{HH}$ = 7.6 Hz, 1H, CH), 6.98 (d, $^3J_{HH}$ = 7.6 Hz, 1H, CH), 6.87 (s, 2H, CH), 6.81 (d, $^3J_{HH}$ = 7.6 Hz, 1H, CH), 6.63 (overlapping signals, 3H, CH), 6.51 (s, 1H, CH), 6.36 (s, 1H, CH), 6.28 (s, 1H, CH), 5.78 (d, $^3J_{HH}$ = 7.6 Hz, 1H, CH), 5.36 (s, 1H, CH), 4.00 (s, 1H, CH), 3.37 (br, 1H, Ar-CH_2Rh), 2.50 (d, $^3J_{HRh}$ = 14.4 Hz, 1H, Ar-CH_2Ge), 2.21 (s, 3H, CH_3 Ar^{Mes}), 2.16 (s, 3H, CH_3 Ar^{Mes}), 2.10 (s, 3H, CH_3 Ar^{Mes}), 2.07 (s, 3H, CH_3 Ar^{Mes}), 2.06 (overlapping signal, 1H, Ar-CH_2Ge), 1.88 (s, 3H, CH_3 Ar^{Mes}), 1.78 (s, 3H, CH_3 Ar^{Mes}), 1.74 (s, 3H, CH_3 Ar^{Mes}), 1.58 (s, 3H, CH_3 Ar^{Mes}), 1.33 (s, 3H, CH_3 Ar^{Mes}), 1.24 (s, 3H, CH_3 Ar^{Mes}), 0.54 (br, 1H, Ar-CH_2Rh).

^{13}C{^{1}H} NMR (100.63 MHz, C_6D_6, 298 K): δ 162.4 (q, $^1J_{CB}$ = 50 Hz, *ipso*-Ar), 152.2 (s, Cq), 148.3 (s, Cq), 146.5 (s, Cq), 142.0 (s, Cq), 141.6 (d, J_{CRh} = 10 Hz, Cq), 141.3 (s, Cq), 141.2 (s, Cq), 138.5 (s, Cq), 138.1 (s, Cq), 137.5 (s, Cq), 136.2 (s, Cq), 136.1 (s, Cq), 136.0 (s, Cq), 135.1 (s, *o*-Ar), 134.3 (s, Cq), 133.8 (s, Cq), 133.2 (d, J_{CRh} = 9 Hz, Cq), 132.1 (s, CH), 131.8 (s, CH), 130.4 (s, CH), 130.0 (s, CH), 129.7 (s, CH), 129.4 (s, CH), 129.1 (s, CH), 129.0 (s, CH), 126.2 (s, Cq), 123.5 (s, Cq), 123.0 (s, Cq), 122.1 (s, CH), 120.8 (s, Cq), 117.7 (s, *p*-Ar), 109.2 (s, Cq), 103.0 (d, J_{CRh} = 6 Hz , Cq), 100.0 (s, CH), 40.6 (d, J_{CRh} = 13 Hz, Ar-CH$_2$Rh), 25.0 (br, Ar-CH$_2$Ge), 23.7 (s, CH$_3$ Ar^{Mes}), 21.6 (s, CH$_3$ Ar^{Mes}), 21.1 (s, CH$_3$ Ar^{Mes}), 20.6 (s, CH$_3$ Ar^{Mes}), 20.5 (s, CH$_3$ Ar^{Mes}), 20.1 (s, CH$_3$ Ar^{Mes}), 18.5 (s, CH$_3$ Ar^{Mes}), 17.2 (s, CH$_3$ Ar^{Mes}), 15.8 (s, CH$_3$ Ar^{Mes}).

2. Solution NMR analysis of compounds of type 2 and 3

As highlighted in the main text, we observed some slight but distinctive NMR spectroscopic differences in compounds **2** and **3** as a result of varying the counteranion. Thus, ^{1}H NMR signals associated to the activated mesityl ring in compounds **2** and **3** containing the triflimidate anion are similar, while differing from the corresponding resonances due to same species when a non-coordinating anion (BAr^F or $[B(C_6F_5)_4]^-$) was used (Figure S1). This would be consistent with triflimidate binding to rhodium accompanied by coordination rearrangement from η^3-pseudoallylic to η^1-methylenic forms. Nonetheless, these differences do not seem to be present in the solid-state, where in all cases η^3-coordination of the flanking aryl ring in a pseudoallylic fashion was elucidated. Also, our prior investigations on a somewhat related cationic gold germylene compound revealed similar effects upon counteranion coordination.[4]

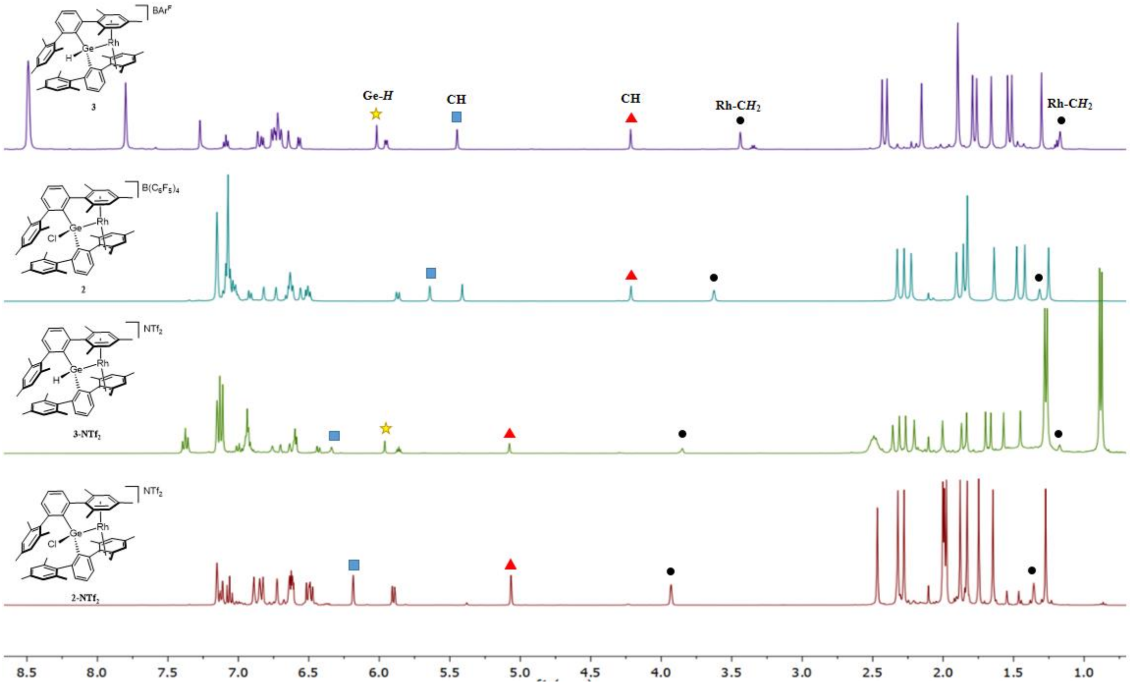

Figure S1. Stacked ^{1}H NMR spectra of compounds of type 2 and 3

3. X-Ray structural characterization of new compounds

Crystallographic details. Low-temperature diffraction data were collected on a Bruker APEX-II CCD diffractometer (**1**) or on a D8 Quest APEX-III single crystal diffractometer with a Photon III detector and a IμS 3.0 microfocus X-ray source (**2**, **3** and **5**) at the Instituto de Investigaciones Químicas, Sevilla. Data were collected by means of ω and φ scans using monochromatic radiation λ(Mo Kα1) = 0.71073 Å. The diffraction images collected were processed and scaled using APEX-II or APEX-III software, respectively. The structures were solved with SHELXT and was refined against F^2 on all data by full-matrix least squares with SHELXL.[5] All non-hydrogen atoms were refined anisotropically. Hydrogen atoms were included in the model at geometrically calculated positions and refined using a riding model, unless otherwise noted. The isotropic displacement parameters of all hydrogen atoms were fixed to 1.2 times the U value of the atoms to which they are linked (1.5 times for methyl groups). Despite many attempts, we could not obtain good quality crystals for compound **3** when pairing with the non-coordinating anion BAr_F ($[B(C_6H_2\text{-}3,5\text{-}(CF_3)_2)_4]^-$). However, we could grow crystals of enough quality for X-ray diffraction studies from the reaction between **1** and two equivalents of $(PMe_2Ar^{Dipp2})Au(NTf_2)$. Thus the structure of **3** contains triflimidate as the counteranion. Similarly, good quality crystals of **2** were obtained after anion exchange by triflate (OTf^-).

The full numbering scheme of all the reported structures can be found in the full details of the X-ray structure determination (CIF), which is included as Supporting Information.

Deposition Number(s) 2083762 (for **1**), 2083760 (for **2**), 2083759 (for **3**), 2083761 (for **5**), contain the supplementary crystallographic data for this paper. These data are provided free of charge by the joint Cambridge Crystallographic Data Centre and Fachinformationszentrum Karlsruhe.

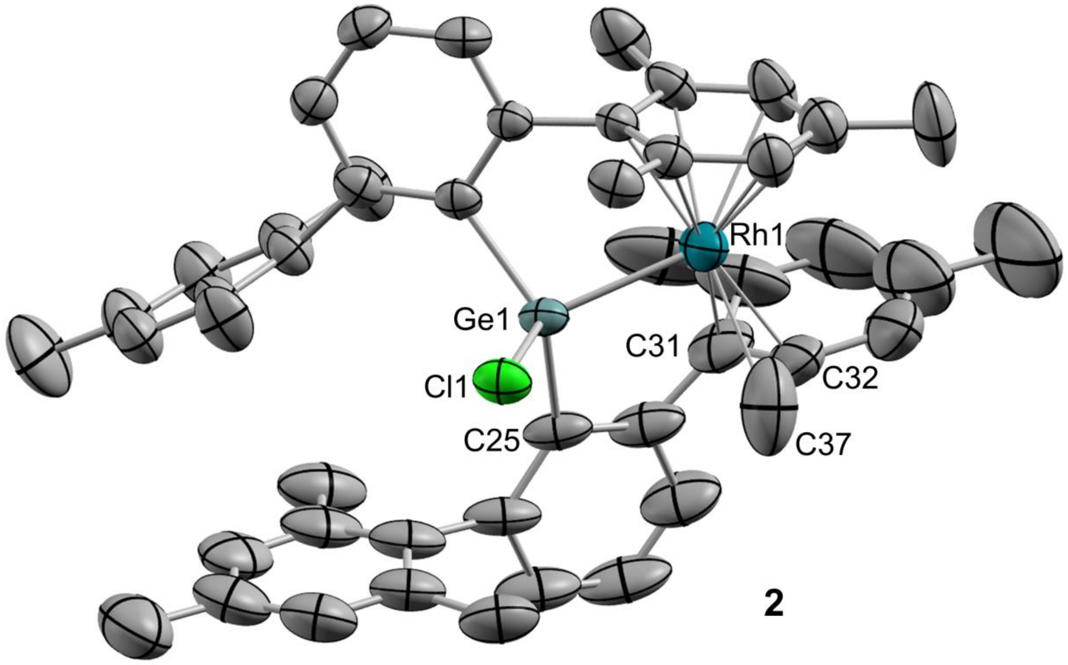

Figure S2. ORTEP diagram of compound **2**. Counteranion, solvent molecules and hydrogen atoms are excluded for clarity. Thermal ellipsoids are set at 50% probability.

Table S1. Crystal data and structure refinement for compounds **1**, **2**, **3** and **5**.

	**1**	**2**	**3**	**5**
Formula	$C_{48}H_{52}ClGeRh$	$C_{58}H_{58}ClF_3GeO_3RhS$	$C_{56}H_{56}F_6GeNO_4RhS_2$	$C_{80}H_{60}BF_{24}GeRh$
Fw	839.84	1103.05	1160.63	1663.59
cryst.size, mm	0.21 × 0.17 × 0.13	0.18 × 0.13 × 0.04	0.10 × 0.09 × 0.03	0.06 × 0.05 × 0.03
crystal system	Monoclinic	Monoclinic	Triclinic	Monoclinic
space group	$P2_1/c$	$P2_1/c$	*P-1*	$P2_1/n$
a, Å	11.3820 (4)	18.0808 (10)	11.9789 (7)	21.1124 (11)
b, Å	15.5958 (6)	18.003 (1)	11.9989 (7)	15.8188 (9)
c, Å	22.3021 (9)	17.1196 (8)	18.8617 (11)	23.3236 (12)
α, deg	90	90	75.067 (2)	90
β, deg	95.959 (1)	111.751 (2)	87.227 (2)	112.895 (2)
γ, deg	90	90	89.740 (2)	90
V, Å^3	3937.5 (3)	5175.8 (5)	2616.3 (3)	7175.8 (7)
T, K	193	193	193	193
Z	4	4	2	4
ρ_{calc}, g cm^{-3}	1.417	1.417	1.473	1.541
μ, mm^{-1} (MoKα)	1.28	1.05	1.04	0.76
F(000)	1736	2272	1188	3356
absorption corrections	multi-scan, 0.60-0.75	multi-scan, 0.63-0.75	multi-scan, 0.60-0.75	multi-scan, 0.67-0.75
θ range, deg	1.6 – 25.3	2.3 – 27.2	2.4 – 28.2	2.1 – 25.3
no. of rflns measd	38659	101254	80384	101525
R_{int}	0.029	0.084	0.051	0.251
no. of rflns unique	7135	11471	9488	12975
no. of params / restraints	472 / 0	609 / 7	641 / 178	974 / 7
R_1 ($I > 2\sigma(I)$) [a]	0.025	0.069	0.086	0.085
R_1 (all data)	0.030	0.119	0.105	0.175
wR_2 ($I > 2\sigma(I)$)	0.069	0.135	0.238	0.206
wR_2 (all data)	0.072	0.174	0.269	0.263
Diff.Fourier.peaks min/max, eÅ^{-3}	-0.87 / 0.41	-2.00 / 2.10	-1.79 / 3.04	-0.98 / 1.61
CCDC number	2083762	2083760	2083759	2083761

4. Computational details

Optimized geometries of Ge—Rh complexes and transition states have been obtained from DFT calculations with Gaussian 09.[6] Optimizations were carried out without symmetry restrictions in bulk solvent (benzene) using DFT methods: the ωB97xD functional[7] was chosen, which includes empirical dispersion corrections[8] and was used in conjunction with the 6-31g(d,p) basis set[9] for non-metal atoms, including Ge, and the SDD basis and associated electron core potential (ECP)[10] for Rh. Bulk solvent effects (benzene) were included during optimization with the SMD continuum model.[11] Topological analysis of the electron density within the Atoms In Molecules (AIM) formalism),[12] and NBO analysis were performed with the Multifwn code[13] and the NBO6.0[14] software respectively. The extended wavefunction [.wfx] and NBO [.47] files required for such analyses were calculated on the geometries previously optimized with the triple-ζ basis set Def2TZVP[15] for all atoms, which includes an ECP for Rh.[16] The CYLview visualization software has been used to create some of the figures.[17]

- DFT-calculated free energy profile in benzene for the formation of species **3**, **4**, and **5** from the cationic germylene-rhodium **A** is shown below (Figure S3). At variance with the profile discussed in the main text, herein intermediates **A'** and **3'** *en route* to **3** are also shown. These intermediates are conformers of **A** and **3**, which result from analysis of the minima connected by **TS$_{A→B}$** and **TS$_{B→3}$** and their geometries can be found in the xyz coordinates file provided.

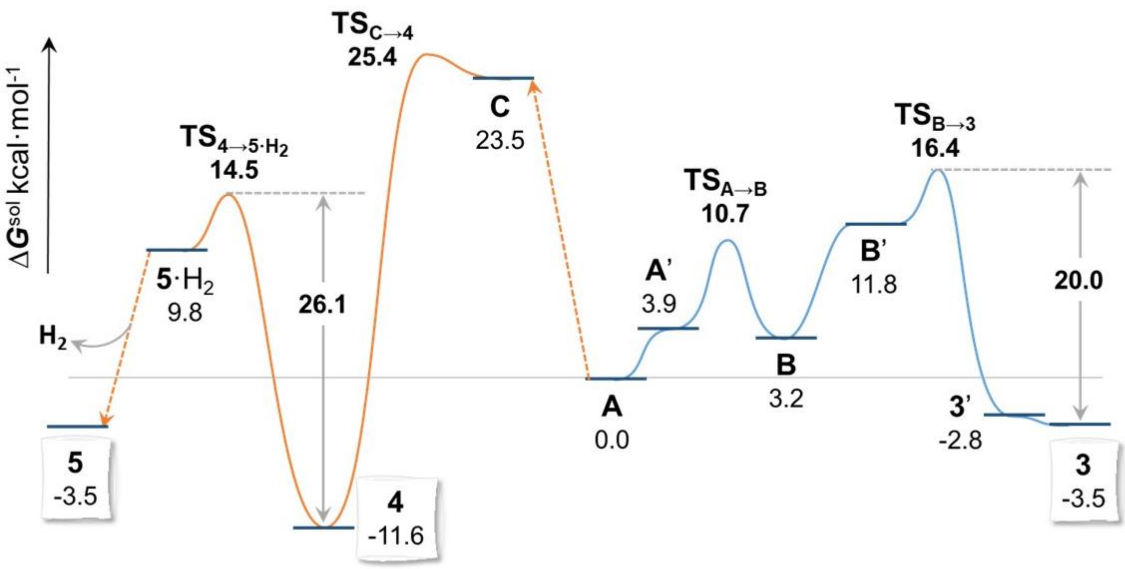

Figure S3. Calculated free energy profile in bulk benzene for the formation of **3**, **4**, and **5**.

Direct formation of **3** *via* C—H activation across the germanium-rhodium bond of **A** was explored and, although a transition state was not located, relaxed

potential energy scans suggest a barrier in excess of 30 kcal·mol^{-1} for this transformation. Similarly, the calculations indicate that direct formation of **4** from **A** and **B** requires overcome barriers of 33.6 and 34.3 kcal·mol^{-1} respectively, which is at odds with the experiments since the formation of **5** from **4** should have a higher energy barrier than the steps leading to **4** from **3**.

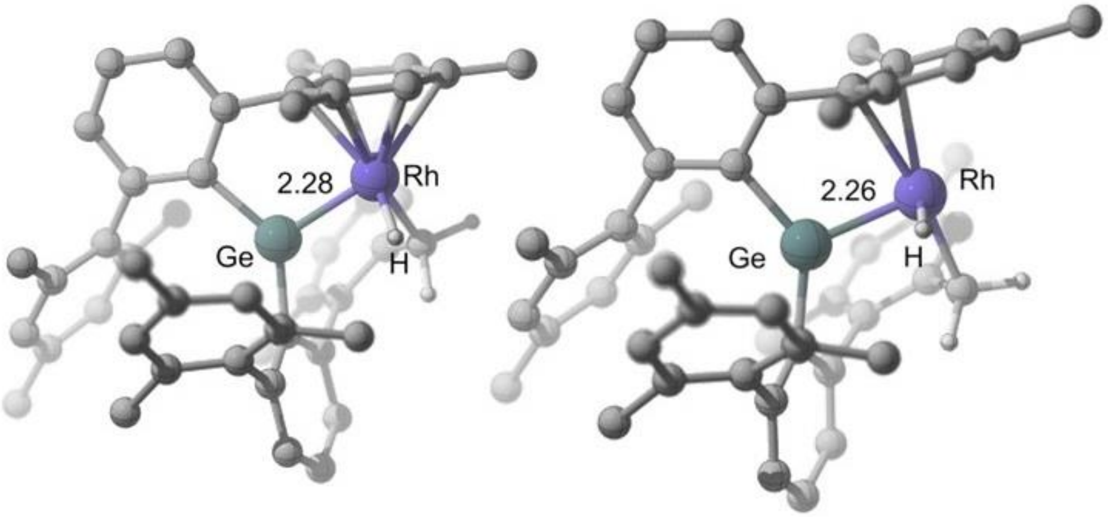

Figure S4. DFT-optimized geometries of **B** and **B'** (most H atoms omitted). Notice the change in the coordination mode of the arene ligand. Further structural parameter can be retrieved from the xyz coordination file provided.

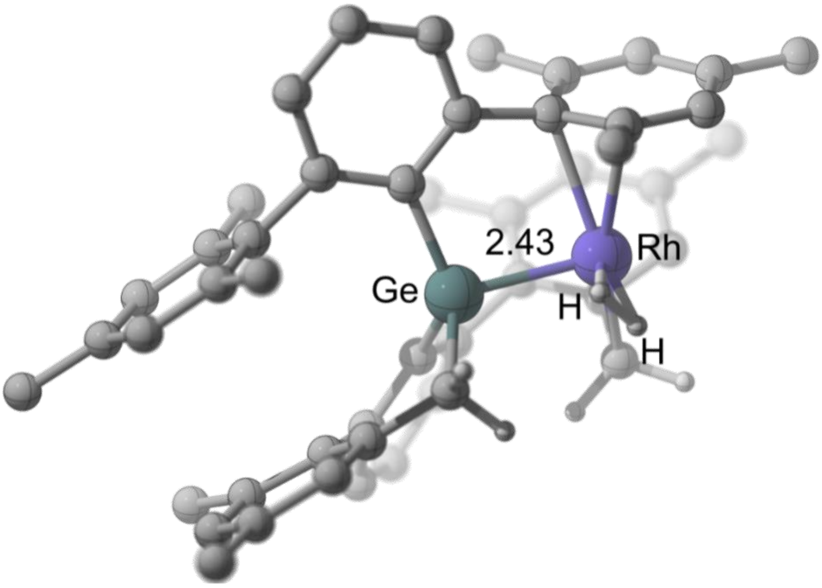

Figure S5. DFT-optimized geometries dihydrogen complex **5·H$_2$** (most H atoms omitted).

- **QTAIM analysis** of the electron density of relevant intermediates and transition states shows bond critical points (bcp) and the corresponding bond paths connecting their germanium and rhodium sites. Figures S6-S11 illustrate these bcps and bond paths overlaid on the Laplacian of the electron density of the corresponding species.

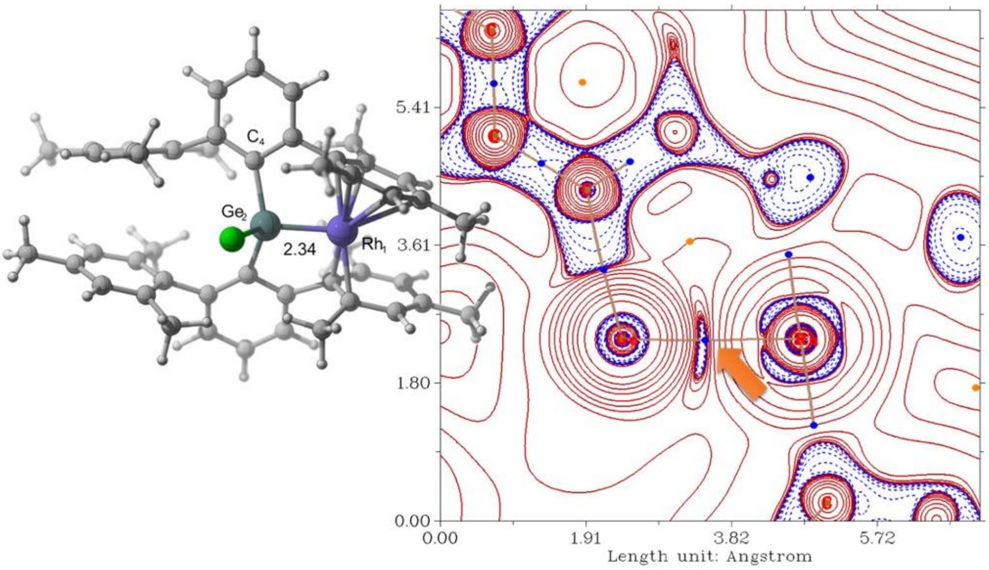

Figure S6. Bond critical points and bond paths of the calculated electron density of **1** on the C4-Ge-Rh plane, overlaid on the Laplacian of the electron density, $\nabla^2\boldsymbol{\rho}$ (solid and dashed lines correspond to positive and negative values respectively). The orange arrow points to the germanium-rhodium bcp.

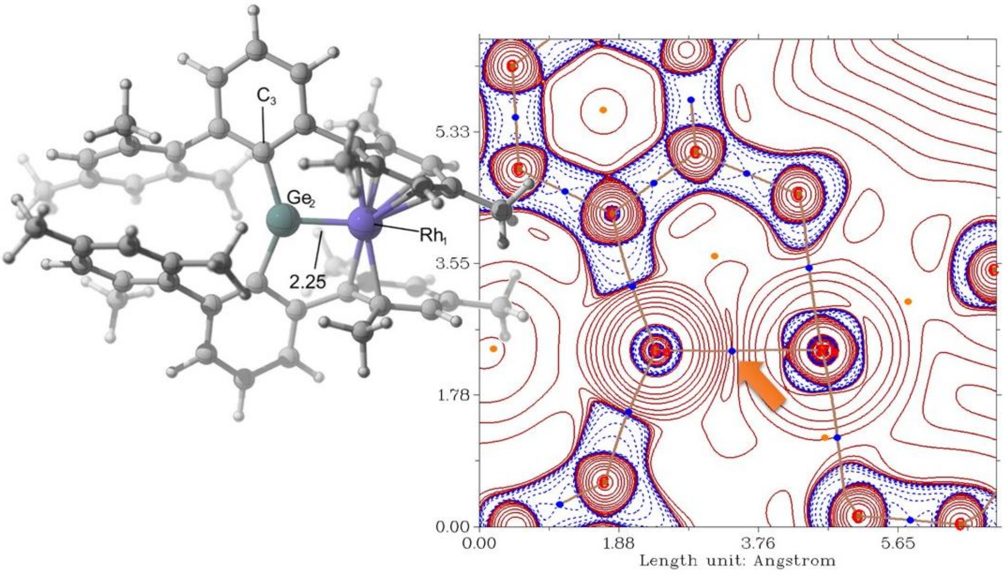

Figure S7. Bond critical points and bond paths of the calculated electron density of **A** on the C3-Ge-Rh plane, overlaid on the Laplacian of the electron density, $\nabla^2\boldsymbol{\rho}$ (solid and

dashed lines correspond to positive and negative values respectively). The orange arrow points to the germanium-rhodium bcp.

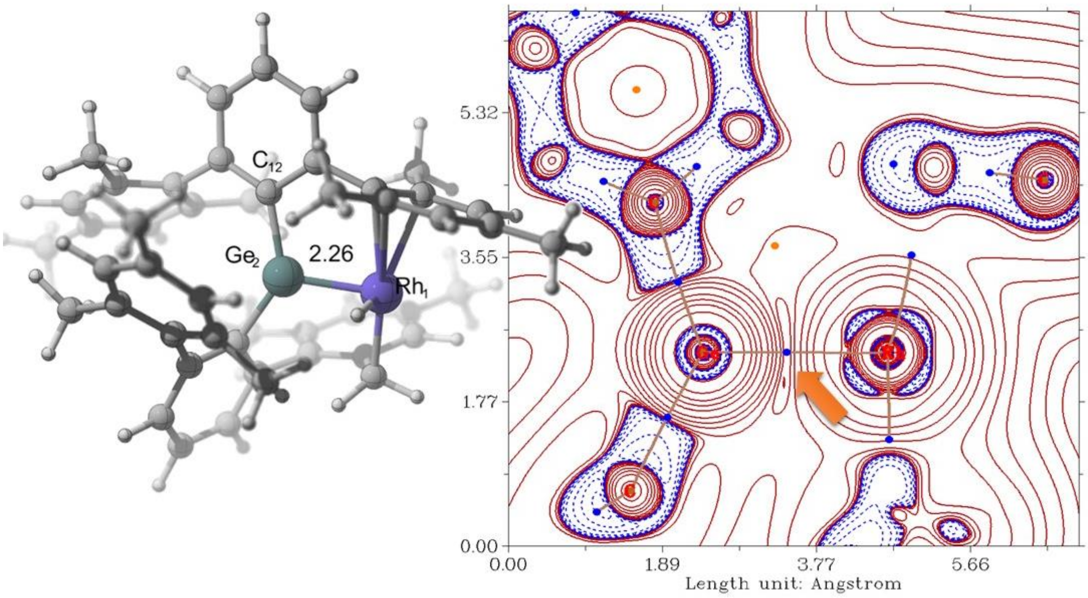

Figure S8. Bond critical points and bond paths of the calculated electron density of **B'** on the C12-Ge-Rh plane, overlaid on the Laplacian of the electron density, $\nabla^2\boldsymbol{\rho}$ (solid and dashed lines correspond to positive and negative values respectively). The orange arrow points to the germanium-rhodium bcp.

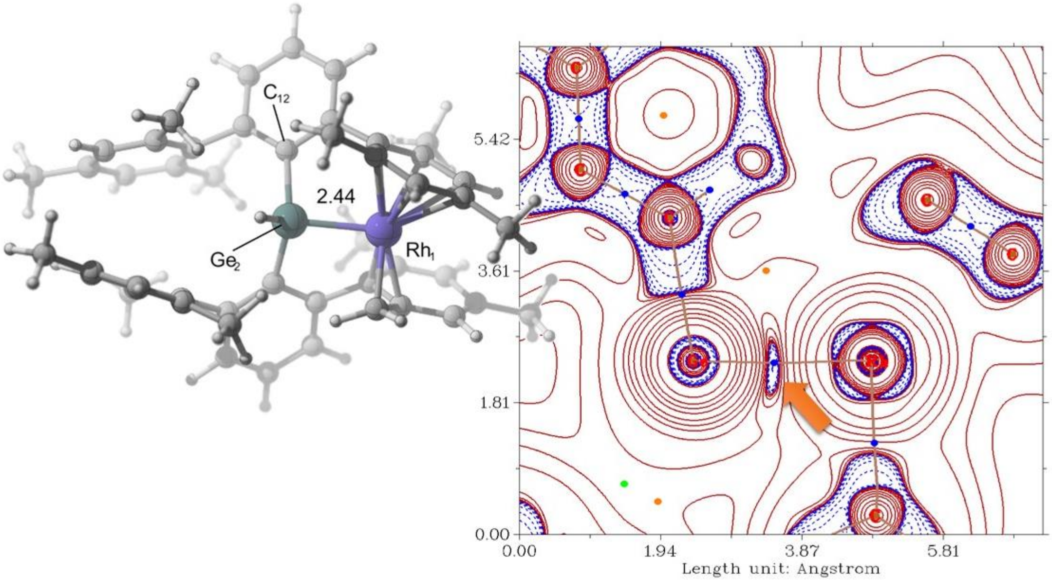

Figure S9. Bond critical points and bond paths of the calculated electron density of **3** on the C3-Ge-Rh plane, overlaid on the Laplacian of the electron density, $\nabla^2\boldsymbol{\rho}$ (solid and dashed lines correspond to positive and negative values respectively). The orange arrow points to the germanium-rhodium bcp.

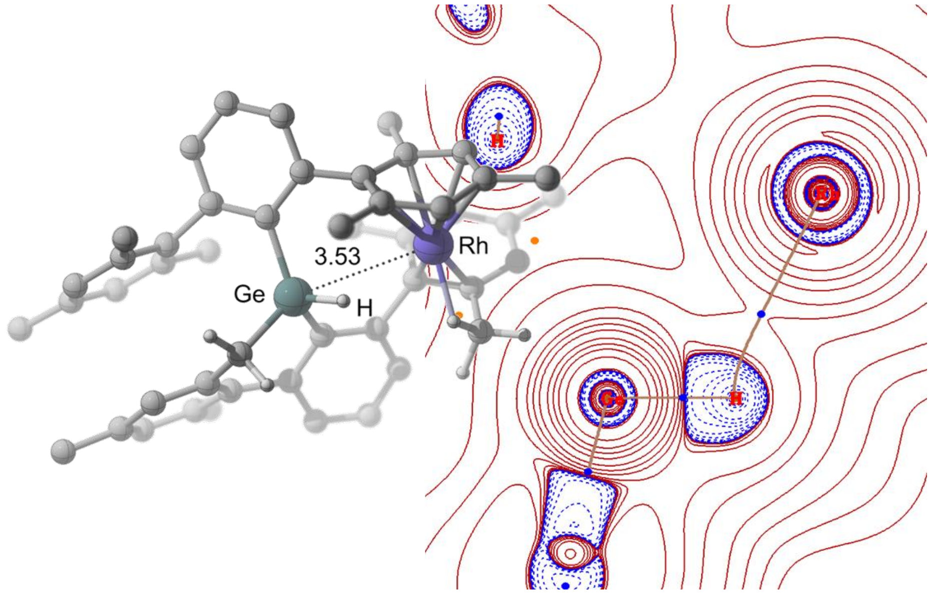

Figure S10. Bond critical points and bond paths of the calculated electron density of **C** on the Ge-H-Rh plane, overlaid on the Laplacian of the electron density, $\nabla^2\boldsymbol{\rho}$ (solid and dashed lines correspond to positive and negative values respectively).

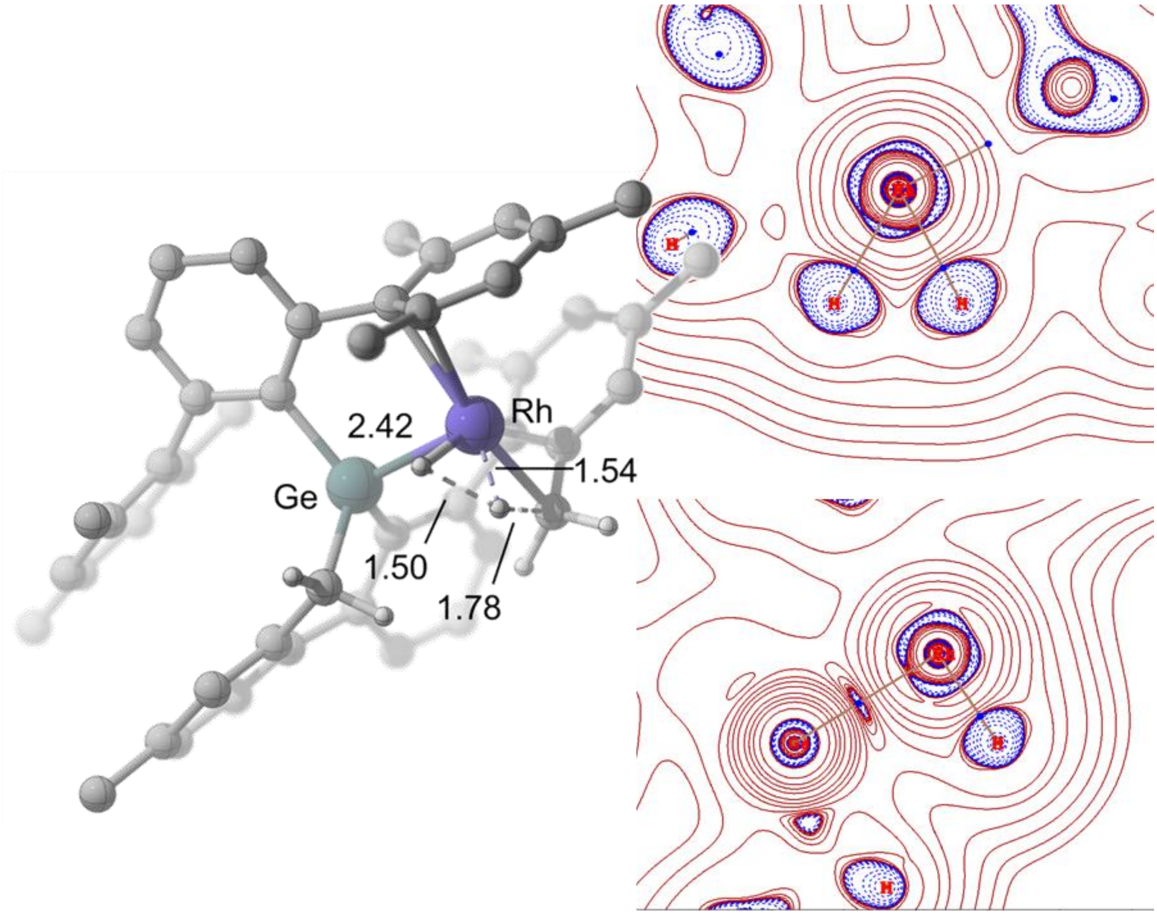

Figure S11. Bond critical points and bond paths of the calculated electron density of **TS$_{4\rightarrow5\cdot H2}$** on the Rh(H-H) plane and on one Ge-H-Rh plane, overlaid on the Laplacian of the electron density, $\nabla^2\boldsymbol{\rho}$ (solid and dashed lines correspond to positive and negative values respectively).

- Analysis of the electron density at the germanium-rhodium bcps of germyl-rhodium species **1** and **3** and germylenes **A** and **B'** (Table S2) reveal electron density values of approximately 0.1 e·bohr^{-3} and values of the Laplacian of their electron densities ($\nabla^2\rho_b$) at the same bcps close to 0. This, in addition to negative values for the total energy density (H_b) and values for the absolute electronic potential energy and kinetic energy densities ratio ($|V_b|/G_b$) close to 2 indicate a strong covalent character for the Ge—Rh interaction in these species.[18] Interestingly, the ellipticity of the Ge—Rh bond increases from 0.09 to ca. 0.2 from the parent germyl **1** to the germylene **A**, which is consistent with an increase in the double bond character (for cylindrical bonds, the ellipticity is expected to be 0). As we shall discuss later, this reflects back-donation from rhodium to germanium that may offset the effect of the loss of the chloride ligand.

Table S2. QTAIM indicators at Ge—Rh BCPs. All data are in atomic units. ρ_b electron density (e·bohr^{-3}); H_b total energy density (hartree·bohr^{-3}); $\nabla^2\rho_b$ Laplacian of the electron density (e·bohr^{-5}); $|V_b|/G_b$ ratio between the absolute electronic potential energy and kinetic energy densities; ε_b ellipticity (ratio between the largest and smallest negative eigenvalues of the Hessian – 1).

Ge—Rh bond	ρ_b	$\nabla^2\rho_b$	G_b	V_b	H_b	$\|V_b\|/G_b$	ε_b
**1**	0.099	-0.005	0.048	-0.0967	-0.049	2.03	0.091
**A**	0.112	0.038	-0.068	-0.127	-0.059	1.86	0.191
**B'**	0.110	0.025	0.064	-0.122	-0.058	1.90	0.077
**$TS_{B'\rightarrow 3}$**	0.094	0.038	0.056	-0.103	-0.047	1.83	0.146
**3**	0.083	-0.018	0.034	-0.072	-0.038	2.13	0.033
**$TS_{4\rightarrow 5\cdot H_2}$**	0.083	-0.009	0.037	-0.077	-0.040	2.056	0.043

- **NBO analysis** was used to complete the insight into the germanium-rhodium interaction of the species studied by AIM. Table S3 summarizes relevant donor-acceptor interactions and Wiberg Bond Order (WBO) of the germanium-rhodium bonds. Typical NBO terminology has been used to name the different types of NBOs: LP for *Lone Pair* and LV for *Lone Vacancy*, which refers to an empty valence orbital localized on one atom. Also, the main Atomic Orbital contribution to the LVs has been included in parenthesis. The NLMO column indicates the percentage of non-Lewis orbitals from the acceptor atom that are mixed with the parent donor NBO.

Table S3. Relevant NBO results showing major donor-acceptor interactions for the Ge—Rh interaction.

Complex	WBO	Donor NBO	Acceptor NBO	ΔE_{ij} kcal·mol^{-1}	NLMO
**A**	0.9043	LP(d) Rh	LV(p) Ge	13.77	2.35% Ge
		LP(d) Rh	LV(p) Ge	26.04	8.04% Ge
**B’**	0.7824	LP(d) Rh	LV(p) Ge	7.63	1.19% Ge
		LP(d) Rh	LV(p) Ge	15.53	3.60% Ge
		BD Rh-H	LV(p) Ge	19.29	4.86% Ge
**3**	0.542	LP(d) Rh	LV(p) Ge	109.57	24.59% Ge
		BD Rh-C_{aryl}	LV(p) Ge	13.44	0.75% Ge
**C**	0.020	-	-	-	-
**$TS_{C\rightarrow4}$**	0.023	-	-	-	-
**$TS_{4\rightarrow5\cdot H2}$**	0.550	LP(d) Rh	LV(p) Ge	63.47	17.97% Ge
		LP(d) Rh	LV(p) Ge	7.42	0.60% Ge

Natural Bonding Orbital (NBO) analysis of the species **1**, **3**, and intermediate **A**, locates 4 *Lone Pairs* (LPs), almost pure d orbitals, on their rhodium atoms, which agrees with a Rh(I)/Ge(II) formulation. Consistent with C—H activation at rhodium, only **3** LPs were located in the case of species **B’**.

Second order perturbation theory analysis of the Fock matrix, reveals electron donation from 4d orbitals of the rhodium atoms to 4p orbitals of the germanium, both in the germyl and germylene species studied, as shown in Table S3. This is supported by the Ge contribution to the corresponding NLMOs.

Notice that the contribution of Ge orbitals to the NLMO corresponding to the donation of electron density from Rh in complex **3** is by far the highest, but it must be recognized that in this case the germanium-rhodium bond is described in terms of electron delocalization (24.59% Ge; ΔE_{ij} = 109.57 kcal·mol^{-1}) between fragments (Figure S12, right).

In complex **B'**, the Ge atom also accepts electron density from the Rh-H bonding orbital, i.e. the bond is delocalized on the germanium (NLMO contribution 4.8%).

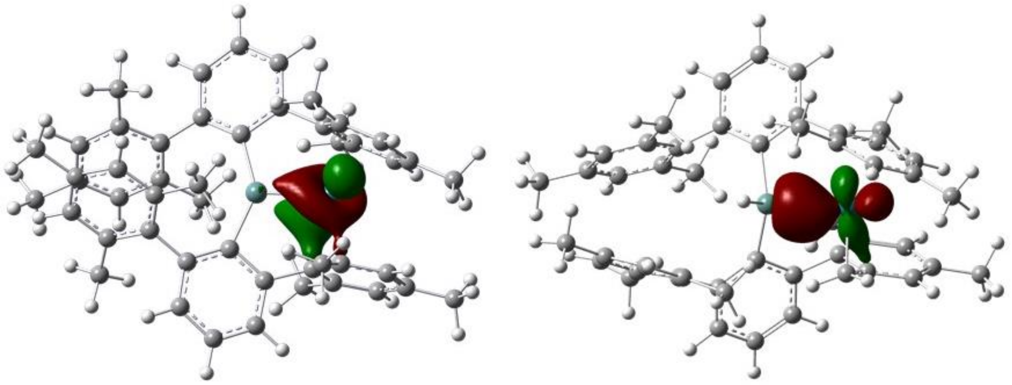

Figure S12. NLMOs involved in the Rh→pGe back donation and in **A** (left) and the germanium-rhodium interaction in **3** (right).

5. NMR spectra of new compounds

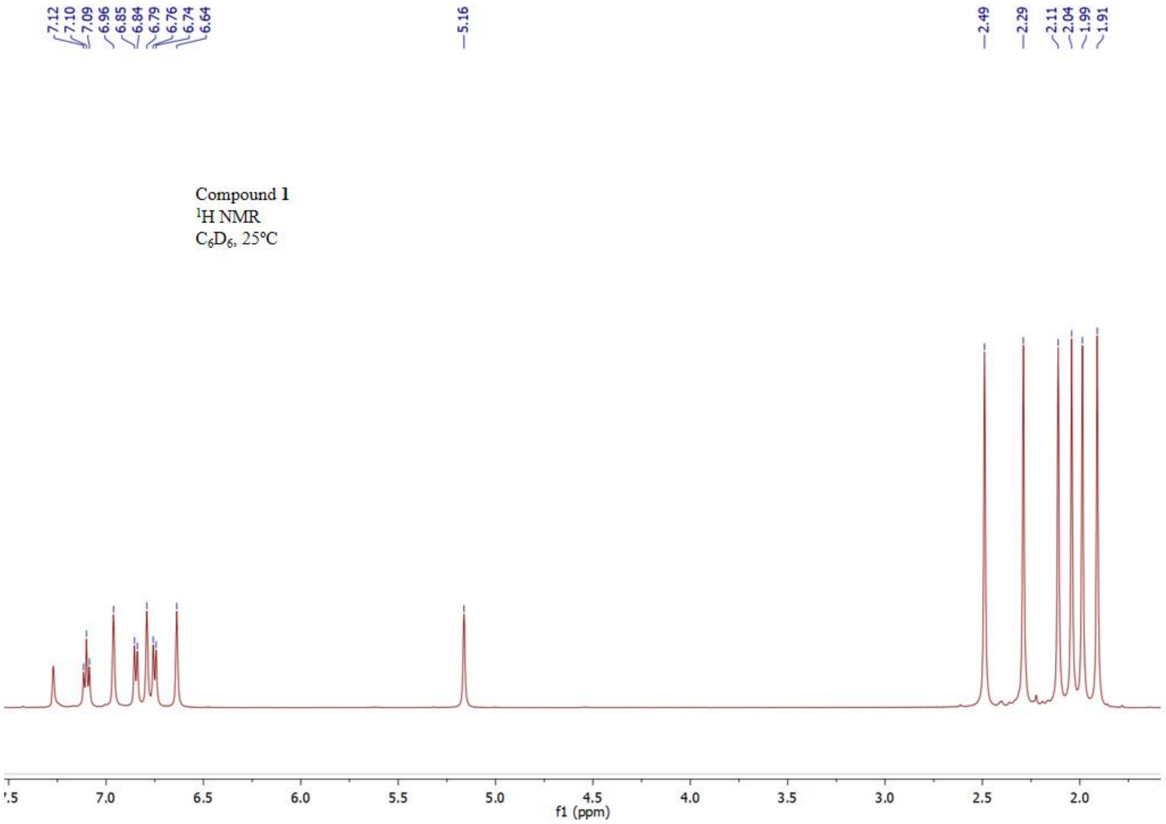

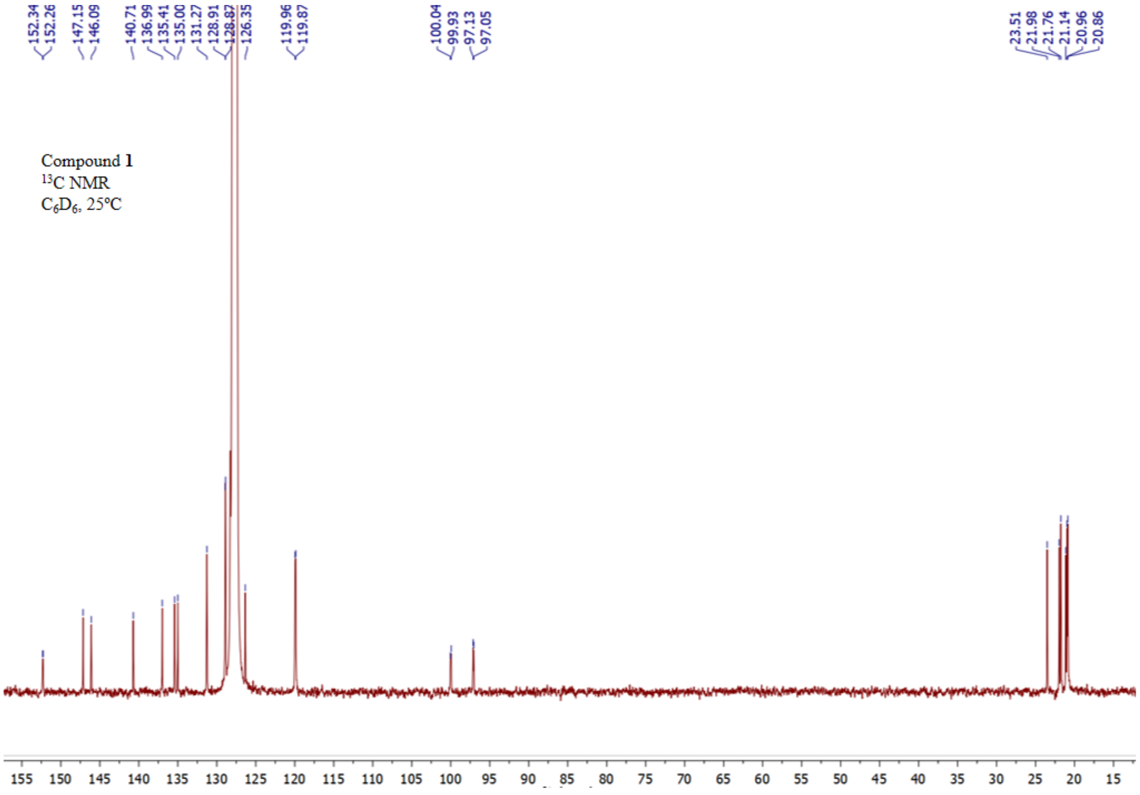

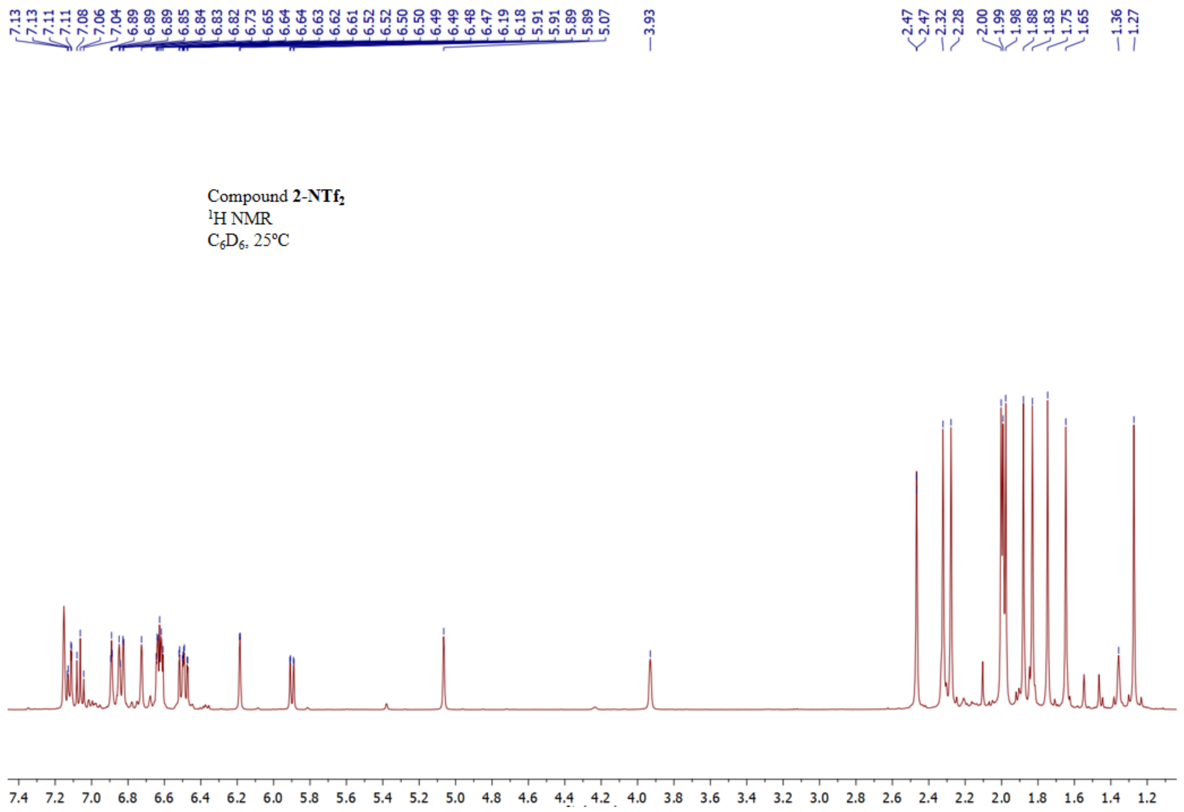
7.13 7.13 7.11 7.11 7.08 7.06 7.04 6.89 6.89 6.89 6.85 6.84 6.83 6.82 6.73 6.65 6.64 6.64 6.63 6.62 6.61 6.52 6.52 6.50 6.50 6.49 6.48 6.47 6.19 6.18 5.91 5.91 5.89 5.89 5.07
3.93
2.47 2.47 2.32 2.28 2.00 1.99 1.98 1.88 1.83 1.75 1.65 1.36 1.27
Compound 2-NTf2
1H NMR
C6D6, 25°C

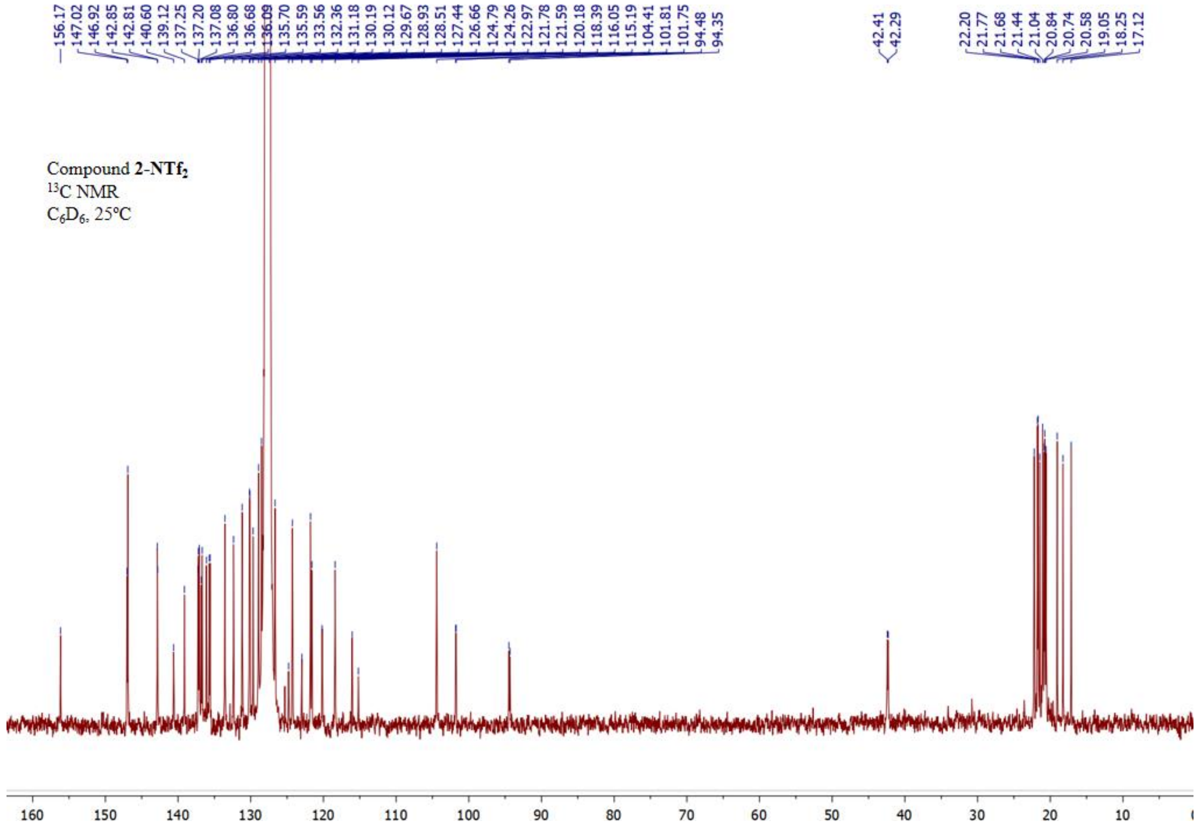
156.17 147.02 146.92 142.85 142.81 140.60 139.12 137.25 137.20 137.08 136.80 136.68 136.09 135.70 135.59 133.56 132.36 131.18 130.19 130.12 129.67 128.93 128.51 127.44 126.66 124.79 124.26 122.97 121.78 121.59 120.18 118.39 116.05 115.19 104.41 101.81 101.75 94.48 94.35
42.41 42.29
22.20 21.77 21.68 21.44 21.04 20.84 20.74 20.58 19.05 18.25 17.12
Compound 2-NTf2
13C NMR
C6D6, 25°C

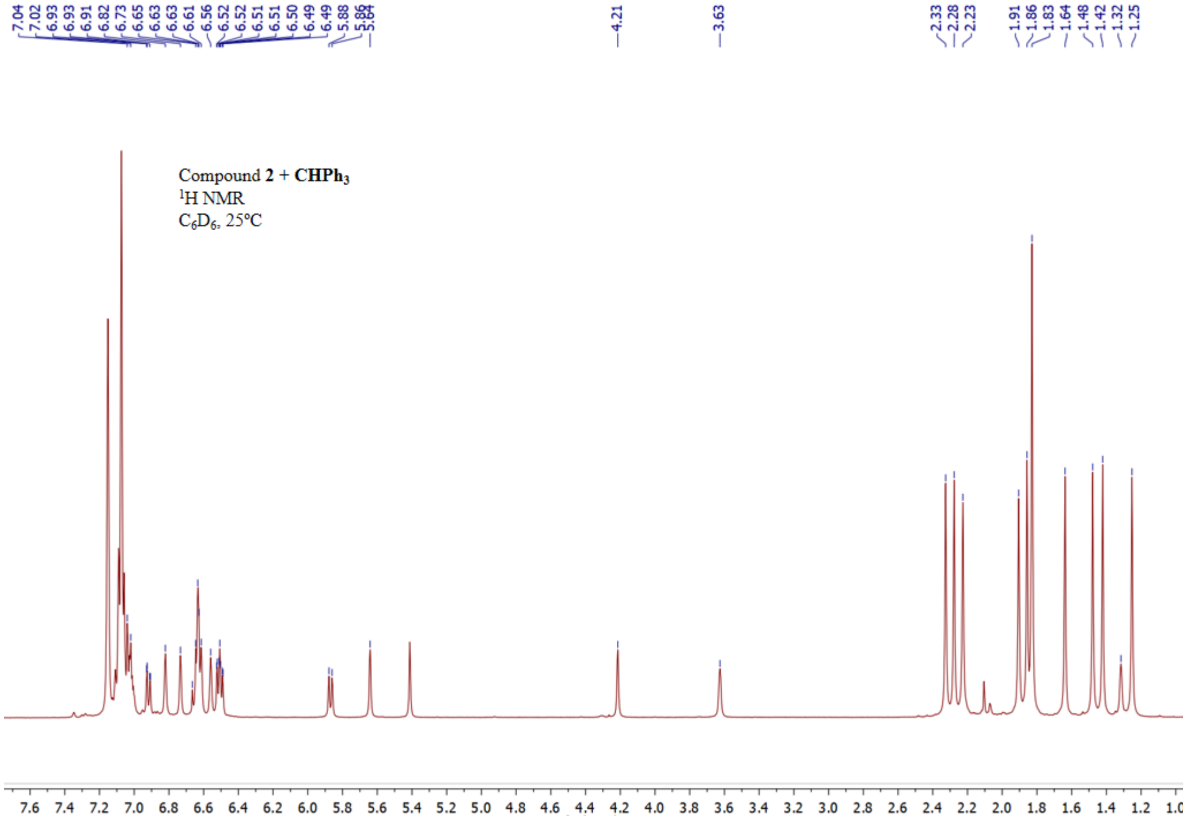
Compound 2 + CHPh3
1H NMR
C6D6, 25°C

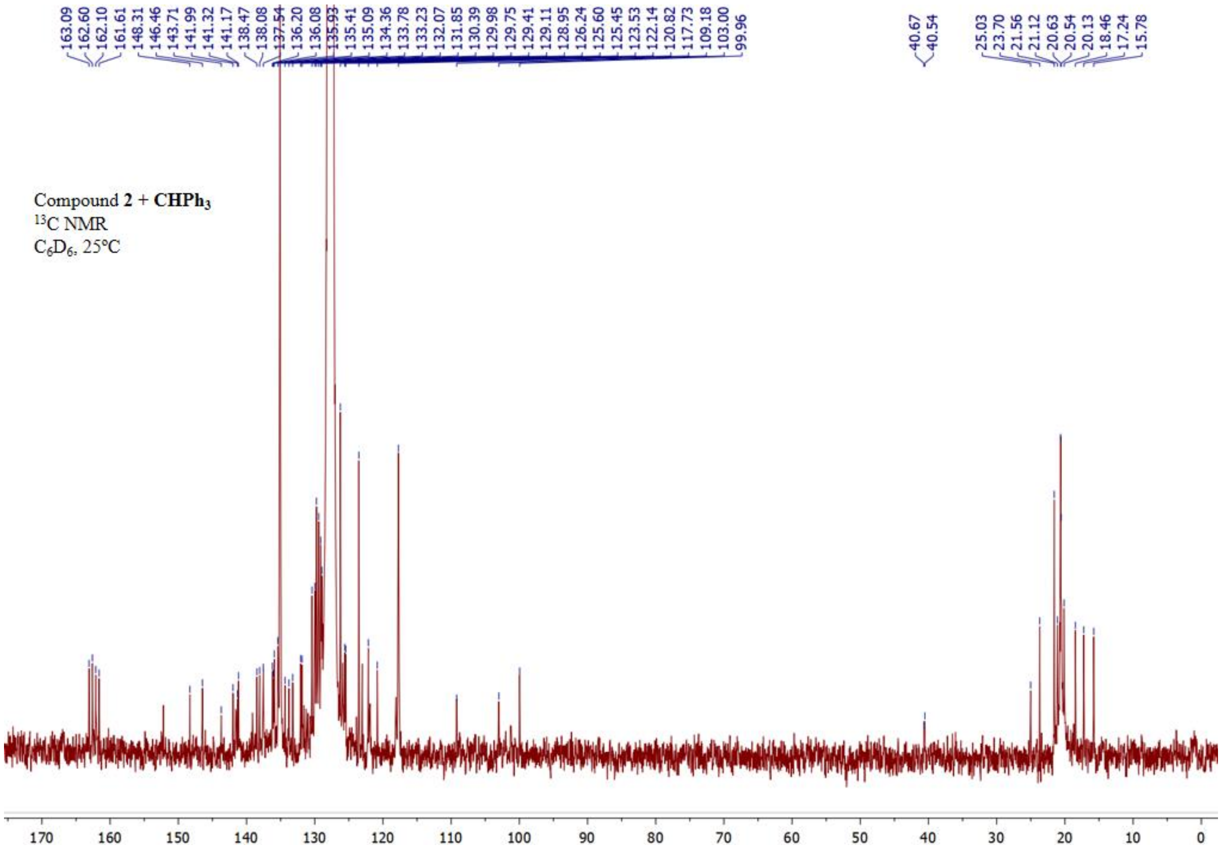
Compound 2 + CHPh3
13C NMR
C6D6, 25°C

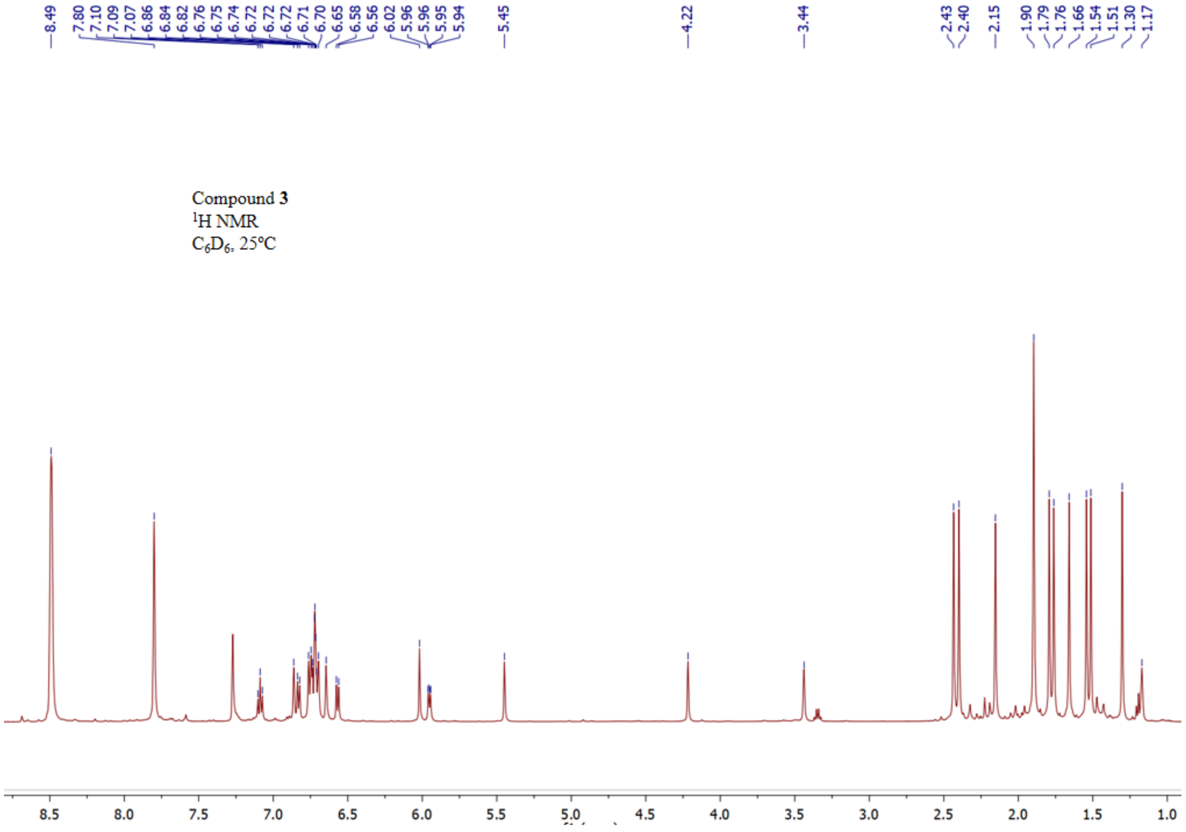
8.49
7.80
7.10
7.09
7.07
6.86
6.84
6.82
6.76
6.75
6.74
6.72
6.72
6.72
6.71
6.70
6.65
6.58
6.56
6.02
5.96
5.96
5.95
5.94
5.45
4.22
3.44
2.43
2.40
2.15
1.90
1.79
1.76
1.66
1.54
1.51
1.30
1.17
Compound 3
1H NMR
C6D6, 25°C
8.5
8.0
7.5
7.0
6.5
6.0
5.5
5.0
4.5
4.0
3.5
3.0
2.5
2.0
1.5
1.0

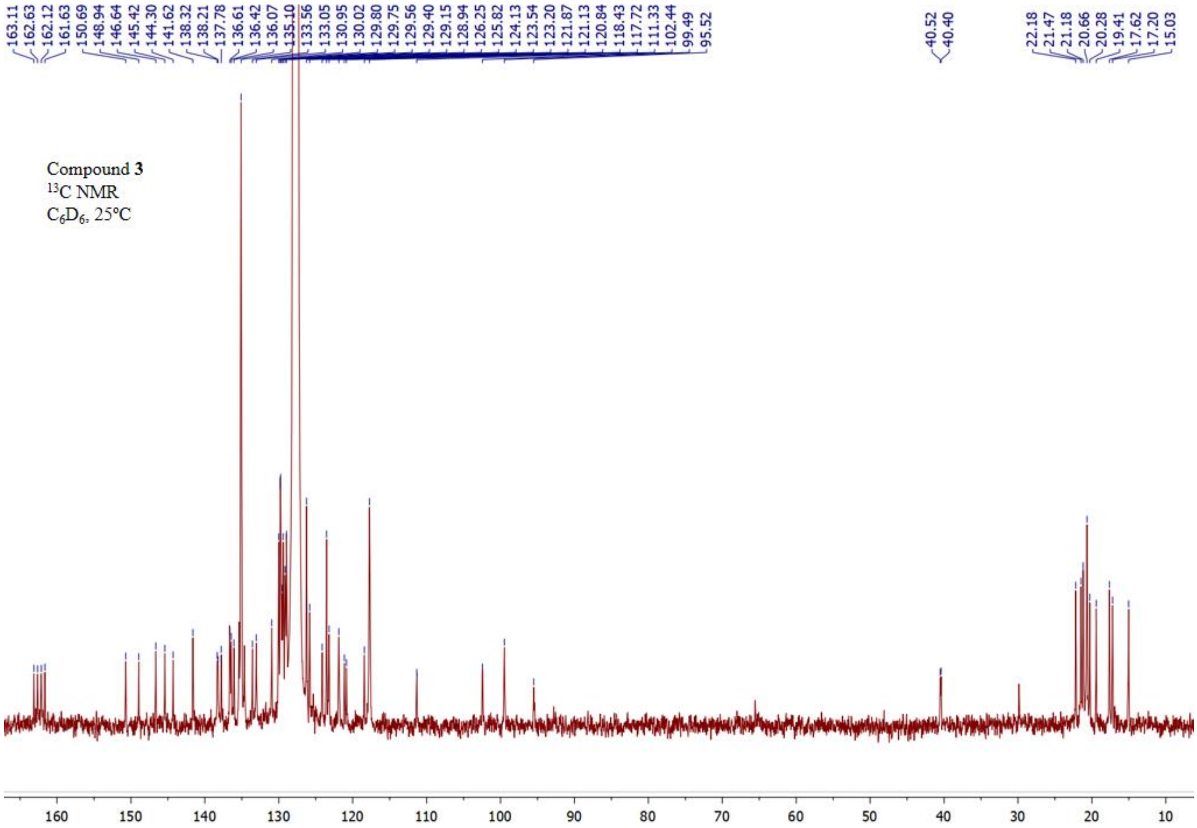
163.11
162.63
162.12
161.63
150.69
148.94
146.64
145.42
144.30
141.62
138.32
138.21
137.78
136.61
136.42
136.07
135.10
133.56
133.05
130.95
130.02
129.80
129.75
129.56
129.40
129.15
128.94
126.25
125.82
124.13
123.54
123.20
121.87
121.13
120.84
118.43
117.72
111.33
102.44
99.49
95.52
40.52
40.40
22.18
21.47
21.18
20.66
20.28
19.41
17.62
17.20
15.03
Compound 3
13C NMR
C6D6, 25°C
160
150
140
130
120
110
100
90
80
70
60
50
40
30
20
10

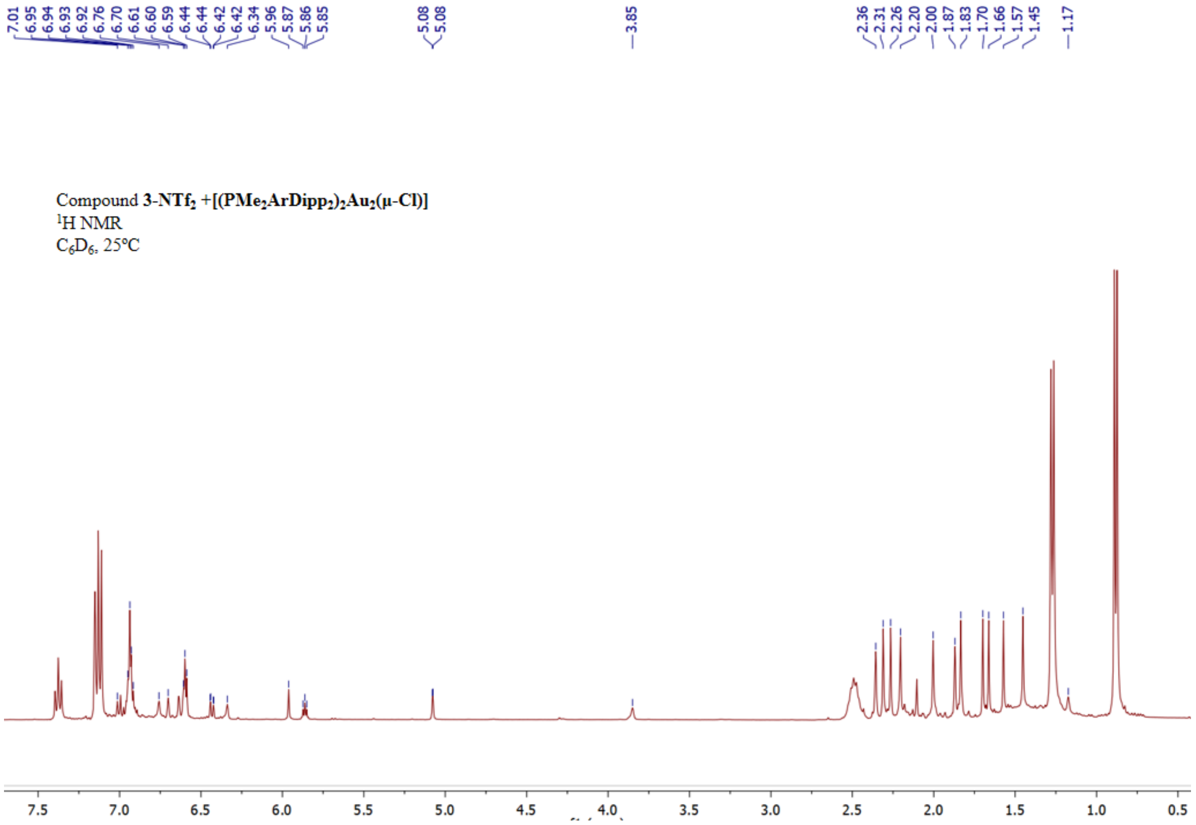
7.01 6.95 6.94 6.93 6.92 6.76 6.70 6.61 6.60 6.59 6.44 6.44 6.42 6.42 6.34 5.96 5.87 5.86 5.85
5.08 5.08
3.85
2.36 2.31 2.26 2.20 2.00 1.87 1.83 1.70 1.66 1.57 1.45
1.17
Compound 3-NTf2 +[(PMe2ArDipp2)2Au2(μ-Cl)]
1H NMR
C6D6, 25°C
7.5 7.0 6.5 6.0 5.5 5.0 4.5 4.0 3.5 3.0 2.5 2.0 1.5 1.0 0.5

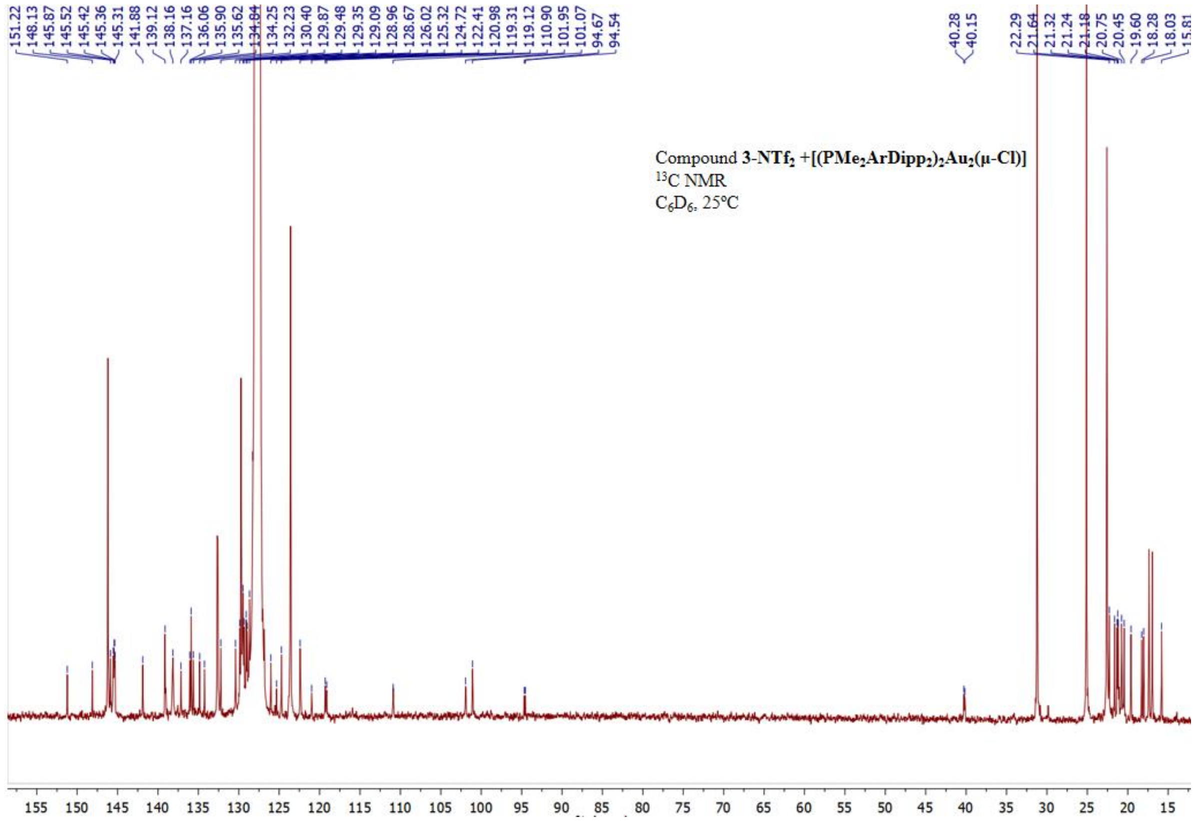
151.22 148.13 145.87 145.52 145.42 145.36 145.31 141.88 139.12 138.16 137.16 136.06 135.90 135.62 134.84 134.25 132.23 130.40 129.87 129.48 129.35 129.09 128.96 128.67 126.02 125.32 124.72 122.41 120.98 119.31 119.12 110.90 101.95 101.07 94.67 94.54
40.28 40.15
22.29 21.64 21.32 21.24 21.18 20.75 20.45 19.60 18.28 18.03 15.81
Compound 3-NTf2 +[(PMe2ArDipp2)2Au2(μ-Cl)]
13C NMR
C6D6, 25°C
155 150 145 140 135 130 125 120 115 110 105 100 95 90 85 80 75 70 65 60 55 50 45 40 35 30 25 20 15

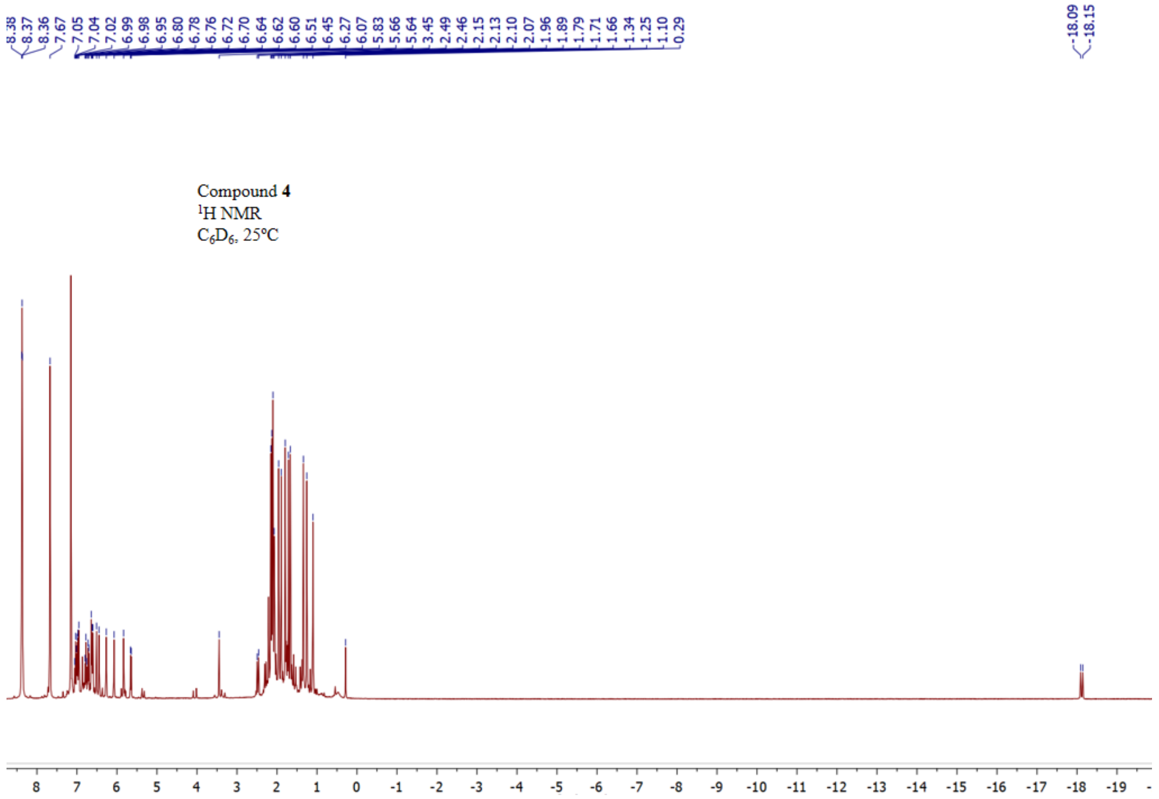
8.38
8.37
8.36
7.67
7.05
7.04
7.02
6.99
6.98
6.95
6.80
6.78
6.76
6.72
6.70
6.64
6.62
6.60
6.51
6.45
6.27
6.07
5.83
5.66
5.64
3.45
2.49
2.46
2.15
2.13
2.10
2.07
1.96
1.89
1.79
1.71
1.66
1.34
1.25
1.10
0.29
-18.09
-18.15
Compound 4
^{1}H NMR
C_6D_6, 25°C
8 7 6 5 4 3 2 1 0 -1 -2 -3 -4 -5 -6 -7 -8 -9 -10 -11 -12 -13 -14 -15 -16 -17 -18 -19

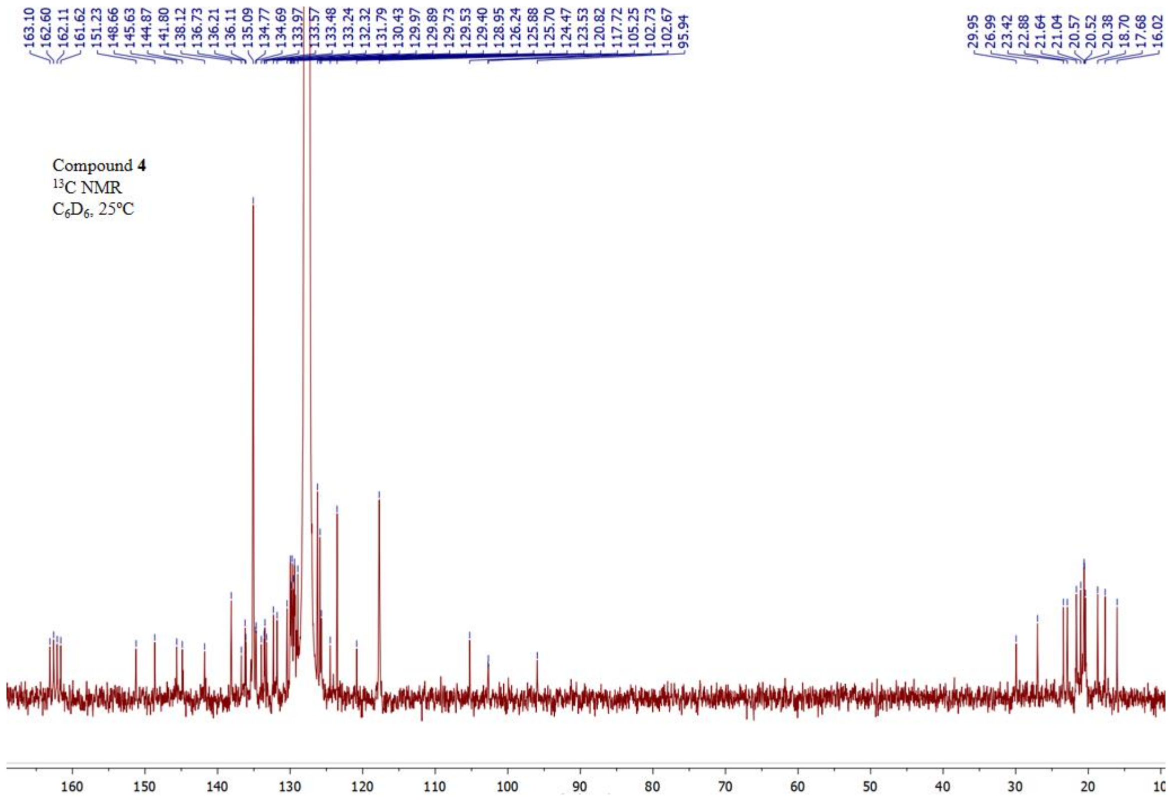
163.10
162.60
162.11
161.62
151.23
148.66
145.63
144.87
141.80
138.12
136.73
136.21
136.11
135.09
134.77
134.69
133.97
133.57
133.48
133.24
132.32
131.79
130.43
129.97
129.89
129.73
129.53
129.40
128.95
126.24
125.88
125.70
124.47
123.53
120.82
117.72
105.25
102.73
102.67
95.94
29.95
26.99
23.42
22.88
21.64
21.04
20.57
20.52
20.38
18.70
17.68
16.02
Compound 4
^{13}C NMR
C_6D_6, 25°C
160 150 140 130 120 110 100 90 80 70 60 50 40 30 20 10

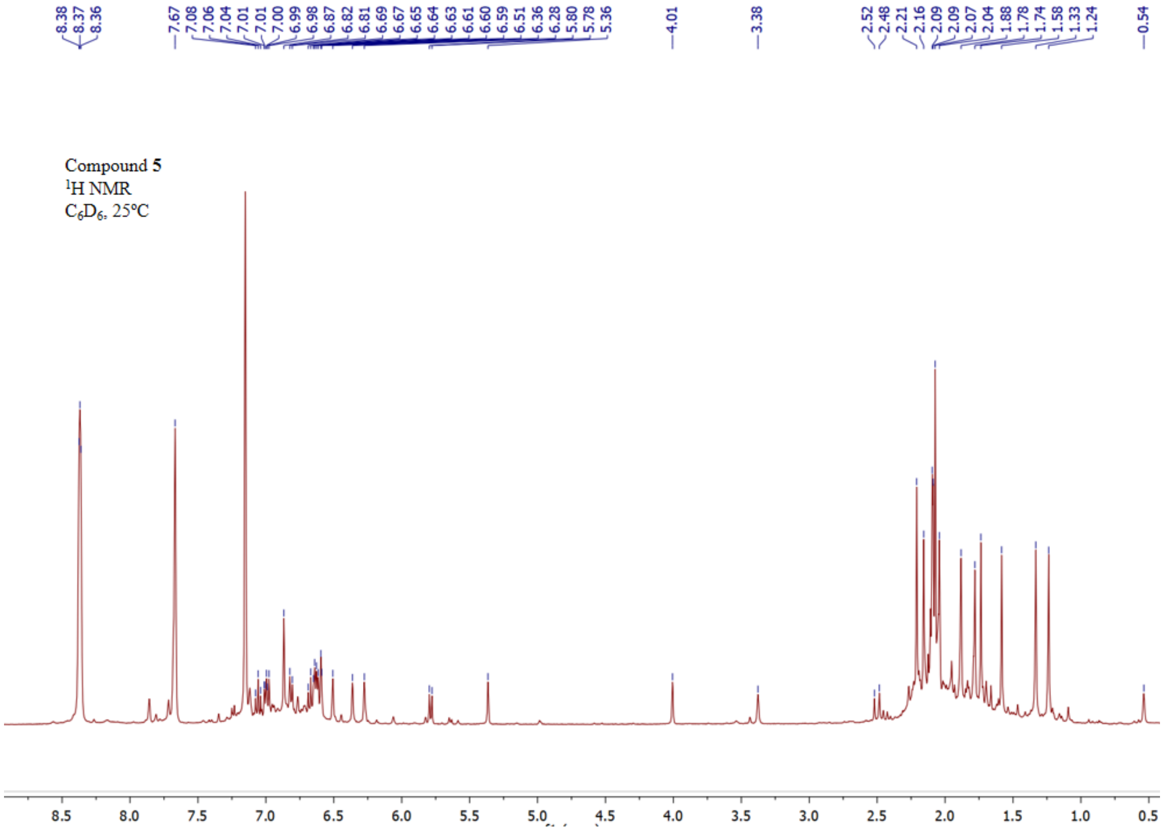
8.38
8.37
8.36
7.67
7.08
7.06
7.04
7.01
7.01
7.00
6.99
6.98
6.87
6.82
6.81
6.69
6.67
6.65
6.64
6.63
6.61
6.60
6.59
6.51
6.36
6.28
5.80
5.78
5.36
4.01
3.38
2.52
2.48
2.21
2.16
2.09
2.09
2.07
2.04
1.88
1.78
1.74
1.58
1.33
1.24
0.54
Compound 5
1H NMR
C6D6, 25°C
8.5
8.0
7.5
7.0
6.5
6.0
5.5
5.0
4.5
4.0
3.5
3.0
2.5
2.0
1.5
1.0
0.5

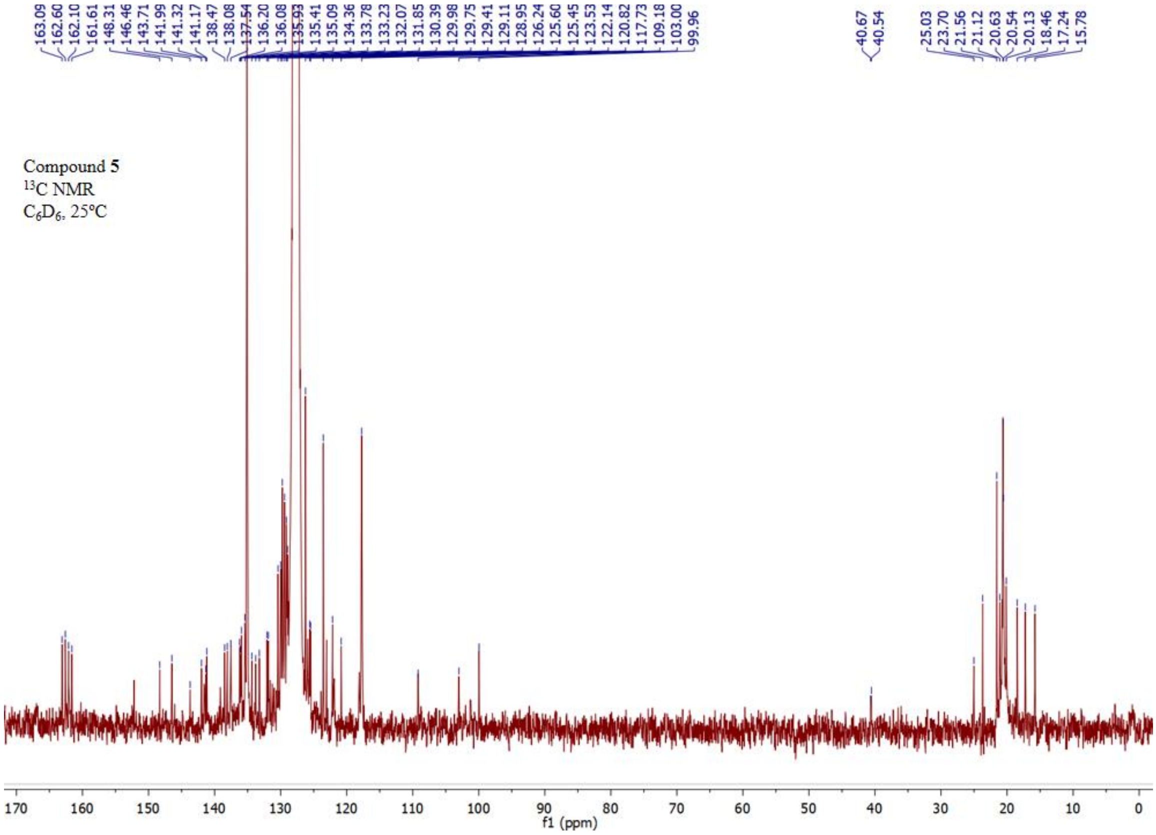
163.09
162.60
162.10
161.61
148.31
146.46
143.71
141.99
141.32
141.17
138.47
138.08
137.54
136.20
136.08
135.93
135.41
135.09
134.36
133.78
133.23
132.07
131.85
130.39
129.98
129.75
129.41
129.11
128.95
126.24
125.60
125.45
123.53
122.14
120.82
117.73
109.18
103.00
99.96
40.67
40.54
25.03
23.70
21.56
21.12
20.63
20.54
20.13
18.46
17.24
15.78
Compound 5
13C NMR
C6D6, 25°C
170
160
150
140
130
120
110
100
90
80
70
60
50
40
30
20
10
0
f1 (ppm)

6. References

1. Simons, R. S.; Pu, L.; Olmstead, M. M.; Power, P. P. Synthesis and Characterization of the Monomeric Diaryls M{C_6H_3-2,6-Mes_2}2 (M = Ge, Sn, or Pb; Mes = 2,4,6-$Me_3C_6H_2$-) and Di-meric Aryl-Metal Chlorides [M(Cl){C_6H_3-2,6-Mes_2}]2 (M = Ge or Sn). *Organometallics* **1997**, *16*, 1920-1925.
2. Giordano, G.; Crabtree, Di-μ-Chloro-Bis(η^4-1,5-Cyclooctadiene)-Dirhodium(I). *Inorg. Synth.* **1979**, *19*, 218.
3. Martínez-Martínez, A. J.; Weller, A. S. Solvent-free anhydrous Li^+, Na^+ and K^+ salts of [B(3,5-$(CF_3)_2C_6H_3)_4$]$^-$, [BArF4]$^-$. Improved synthesis and solid-state structures. *Dalton Trans.* **2019**, *48*, 3551-3554.
4. Bajo, S.; Alcaide, M. M.; López-Serrano, J.; Campos, J. Structural Snapshots of π-Arene Bonding in a Gold Germylene Cation. *Chem. Eur.J.* **2020**, *26*, 15519 –15523.
5. G. M. Sheldrick, *Acta Cryst*. **2008,** *A64*, 112–122.
6. Gaussian 09, Revisions E.01 and B.01, M. J. Frisch, G. W. Trucks, H. B. Schlegel, G. E. Scuseria, M. A. Robb, J. R. Cheeseman, G. Scalmani, V. Barone, B. Mennucci, G. A. Petersson, H. Nakatsuji, M. Caricato, X. Li, H. P. Hratchian, A. F. Izmaylov, J. Bloino, G. Zheng, J. L. Sonnenberg, M. Hada, M. Ehara, K. Toyota, R. Fukuda, J. Hasegawa, M. Ishida, T. Nakajima, Y. Honda, O. Kitao, H. Nakai, T. Vreven, J. A. Montgomery, Jr., J. E. Peralta, F. Ogliaro, M. Bearpark, J. J. Heyd, E. Brothers, K. N. Kudin, V. N. Staroverov, T. Keith, R. Kobayashi, J. Normand, K. Raghavachari, A. Rendell, J. C. Burant, S. S. Iyengar, J. Tomasi, M. Cossi, N. Rega, J. M. Millam, M. Klene, J. E. Knox, J. B. Cross, V. Bakken, C. Adamo, J. Jaramillo, R. Gomperts, R. E. Stratmann, O. Yazyev, A. J. Austin, R. Cammi, C. Pomelli, J. W. Ochterski, R. L. Martin, K. Morokuma, V. G. Zakrzewski, G. A. Voth, P. Salvador, J. J. Dannenberg, S. Dapprich, A. D. Daniels, O. Farkas, J. B. Foresman, J. V. Ortiz, J. Cioslowski, and D. J. Fox, Gaussian, Inc., Wallingford CT, 2010.
7. J.-D. Chai and M. Head-Gordon, "Long-range corrected hybrid density functionals with damped atom-atom dispersion corrections," *Phys. Chem. Chem. Phys.,* 10 (**2008**) 6615-20. DOI: 10.1039/B810189B
8. S. Grimme, "Semiempirical GGA-type density functional constructed with a long-range dispersion correction," *J. Comp. Chem.*, 27 (**2006**) 1787-99. DOI: 10.1002/jcc.20495
9. (a) Ditchfield, R.; Hehre, W. J.; Pople. J. A. Self-Consistent Molecular-Orbital Methods. IX. An Extended Gaussian-Type Basis for Molecular-Orbital Studies of Organic Molecules. *J. Chem. Phys.* **1971**, *54*, 724–728; (b) Hehre, W. J.; Ditchfield, R.; Pople J. A. Self—Consistent Molecular Orbital Methods. XII. Further Extensions of Gaussian-Type Basis Sets for Use in Molecular Orbital Studies of Organic Molecules. *J. Chem.*

Phys. **1972**, *56*, 2257–2261; (c) Hariharan, P. C.; Pople, J. A. The Influence of Polarization Functions on Molecular Orbital Hydrogenation Energies. Theor. *Chim. Acta* **1973**, *28*, 213–222; (d) Francl, M. M.; Pietro, W. J.; Hehre, W. J.; Binkley, J. S.; Gordon, M. S.; DeFrees, D. J.; Pople. J. A. Self-Consistent Molecular Orbital Methods. XXIII. A Polarization-Type Basis Set for Second-Row Elements. *J. Chem. Phys.* **1982**, *77*, 3654–3665.

10. Andrae, D.; Haeussermann, U.; Dolg, M.; Stoll, H.; Preuss, H. Energy-Adjusted Ab Initio Pseudopotentials for the Second and Third Row Transition Elements, Theor. *Chim. Acta* **1990**, *77*, 123–141.
11. Marenich, A. V.; Cramer, C. J.; Truhlar, D. G. Universal Solvation Model Based on Solute Electron Density and on a Continuum Model of the Solvent Defined by the Bulk Dielectric Constant and Atomic Surface Tensions, *J. Phys. Chem.* B **2009**, *113*, 6378–6396.
12. (a) Bader, R. F. W. Atoms in Molecules: A Quantum Theory, Oxford University Press, Oxford, 1995; (b) Bader, R. F. W. A Quantum Theory of Molecular Structure and Its Applications, *Chem. Rev.* **1991**, *91*, 893-928.
13. (a) Multiwfn, v. 6.0. http://sobereva.com/multiwfn/; (b) Lu, T.; Chen, F. Multiwfn: A multifunctional wavefunction analyzer. *J. Comput. Chem.* **2012**, *33*, 580-592.
14. (a) Glendening, E. D.; Landis, C. R.; Weinhold, F. NBO 6.0 : Natural bond orbital analysis program. *J. Comput. Chem.* **2013**, *34*, 1429-1437; (b) Glendening, E. D.; Badenhoop, J. K.; Reed, A. E.; Carpenter, J. E.; Bohmann, J. A.; Morales, C. M.; Landis, C. R.; Weinhold, F. NBO 6.0.; Theoretical Chemistry Institute, University of Wisconsin: Madi-son, 2013. Available at: www.chem.wisc.edu.
15. Weigend, F.; Ahlrichs, R. Balanced basis sets of split valence, triple zeta valence and quadruple zeta valence quality for H to Rn: Design and assessment of accuracy, *Phys. Chem. Chem. Phys.* **2005**, *7*, 329.
16. Andrae, D., Häußermann, U., Dolg, M., Stoll, H., Preuß, H., Energy-adjusted initio pseudopotentials for the second and third row transition elements, *Theor. Chim. Acta*, **1990**, *77*, 123-141.
17. CYLview, 1.0b; Legault, C. Y., Université de Sherbrooke, 2009 (http://www.cylview.org).
18. (a) P. L. A. Popelier, The QTAIM Perspective of Chemical Bonding. In The Chemical Bond, Eds. G. Frenking, S. Shaik, 2014; b) E. Espinosa, I. Alkorta, J. Elguero, E. Molins, *J. Chem. Phys.* **2002**, *117*, 5529 – 5542.
